# Arsenolipids in Cultured *Picocystis* Strain ML and Their Occurrence in Biota and Sediment from Mono Lake, California

**DOI:** 10.3390/life10060093

**Published:** 2020-06-24

**Authors:** Ronald A. Glabonjat, Jodi S. Blum, Laurence G. Miller, Samuel M. Webb, John F. Stolz, Kevin A. Francesconi, Ronald S. Oremland

**Affiliations:** 1 Institute of Chemistry, NAWI Graz, University of Graz, 8010 Graz, Austria; ronald.glabonjat@uni-graz.at (R.A.G.); kevin.francesconi@uni-graz.at (K.A.F.); 2 Water Mission Area, US Geological Survey, Menlo Park, CA 94025, USA; jsblum@usgs.gov (J.S.B.); lgmiller@usgs.gov (L.G.M.); 3 Stanford Synchrotron Radiation Lightsource, Menlo Park, CA 94025, USA; samwebb@slac.stanford.edu; 4 Department of Biological Sciences, Duquesne University, Pittsburgh, PA 15282, USA; stolz@duq.edu

**Keywords:** organo-arsenic, arsenolipids, picoplankton, soda lakes

## Abstract

Primary production in Mono Lake, a hypersaline soda lake rich in dissolved inorganic arsenic, is dominated by *Picocystis* strain ML. We set out to determine if this photoautotrophic picoplankter could metabolize inorganic arsenic and in doing so form unusual arsenolipids (e.g., arsenic bound to 2-*O*-methyl ribosides) as reported in other saline ecosystems and by halophilic algae. We cultivated *Picocystis* strain ML on a seawater-based medium with either low (37 µM) or high (1000 µM) phosphate in the presence of arsenite (400 µM), arsenate (800 µM), or without arsenic additions (ca 0.025 µM). Cultivars formed a variety of organoarsenic compounds, including a phytyl 2-*O*-methyl arsenosugar, depending upon the cultivation conditions and arsenic exposure. When the cells were grown at low P, the organoarsenicals they produced when exposed to both arsenite and arsenate were primarily arsenolipids (~88%) with only a modest content of water-soluble organoarsenic compounds (e.g., arsenosugars). When grown at high P, sequestration shifted to primarily water-soluble, simple methylated arsenicals such as dimethylarsinate; arsenolipids still constituted ~32% of organoarsenic incorporated into cells exposed to arsenate but < 1% when exposed to arsenite. Curiously, *Picocystis* strain ML grown at low P and exposed to arsenate sequestered huge amounts of arsenic into the cells accounting for 13.3% of the dry biomass; cells grown at low P and arsenite exposure sequestered much lower amounts, equivalent to 0.35% of dry biomass. Extraction of a resistant phase with trifluoroacetate recovered most of the sequestered arsenic in the form of arsenate. Uptake of arsenate into low P-cultivated cells was confirmed by X-ray fluorescence, while XANES/EXAFS spectra indicated the sequestered arsenic was retained as an inorganic iron precipitate, similar to scorodite, rather than as an As-containing macromolecule. Samples from Mono Lake demonstrated the presence of a wide variety of organoarsenic compounds, including arsenosugar phospholipids, most prevalent in zooplankton (*Artemia*) and phytoplankton samples, with much lower amounts detected in the bottom sediments. These observations suggest a trophic transfer of organoarsenicals from the phytoplankton (*Picocystis*) to the zooplankton (*Artemia*) community, with efficient bacterial mineralization of any lysis-released organoarsenicals back to inorganic oxyanions before they sink to the sediments.

## 1. Introduction

The presence of organoarsenic in marine organisms was first reported almost 100 years ago in the classic studies of Jones [1] and Chapman [2], and today, it is well known that many organoarsenic species occur naturally at high concentrations in marine organisms [3,4]. Over 200 organoarsenic species have been described with arsenosugars (AsSug), the most dominant species in marine algae, whereas arsenobetaine (AB) represents the prominent species in most marine animals [5]. The first lipid-soluble As (arsenolipid) species were identified later, in 1988 by Morita and Shibata [6], but since then, marine organisms have proven to be a rich source of various types of arsenolipids (As-lipids). These include fatty acids [7,8], hydrocarbons [8,9], fatty alcohols [10], phospholipids [11,12,13,14], di- and tri-acylglycerols [14], and an unusual ether-bound phytyl 2-*O*-methyl arsenosugar [15].

Several studies have contributed to our understanding of the occurrence of arsenolipids [16,17,18,19,20,21,22], but we still have only limited knowledge about the metabolism of these compounds and their behavior in natural systems. In this regard, two recent studies by Glabonjat et al. [19,22] have revealed the likely significance of phytyl 2-*O*-methyl arsenosugars in the natural cycling of arsenic. In culture experiments, the unicellular alga *Dunaliella tertiolecta* transformed arsenate primarily into arsenoribosides including phytyl 2-*O*-methyl arsenosugar, and in a field study in the Great Salt Lake, an extreme hypersaline environment, this compound was a significant organoarsenical in sediments, its likely origin being planktonic *Dunaliella* species that are dominant in the overlying water body.

Further advances in our understanding of arsenic cycling await studies that can directly compare results from field observations with controlled laboratory experiments. Guided by the observations of Glabonjat et al. and hypothesizing the significance of ether-bound isoprenoyl 2-*O*-methyl arsenosugars, we sought a halotolerant/halophilic phytoplanktonic organism that might be abundant in extreme environments and in addition would be amenable to culture experiments. Here, we report an investigation using high-performance liquid chromatography (HPLC)–mass spectrometry and X-ray spectroscopic methods to examine the arsenic species in cultured *Picocystis* strain ML as well as in collected samples representing a basic food web in Mono Lake, California, USA.

## 2. Materials and Methods

### 2.1. Research Site 

Mono Lake is a closed basin soda lake located in central, eastern California on the edge of the Great Basin Desert. In contrast to the pH-neutral Great Salt Lake, Mono Lake is alkaline (pH = 9.8), moderately hypersaline (salinity ~80–90 g/L), and contains abundant dissolved inorganic arsenic (~200 µM) [23] and inorganic phosphate (~1,000 µM) [24]. When monomictic, the lake supports high levels (499–641 g/m^2^) of annual primary production [24,25]. The most conspicuous and abundant member of the phytoplankton community is *Picocystis* strain ML, a eukaryotic picoplankter (diameter ~3 µm). Strain ML is responsible for at least 50% of the lake’s primary productivity, is distributed throughout Mono Lake’s water column, is readily adapted to low light levels and the chemically harsh reducing conditions of the lake below the oxycline, and is grazed down by brine shrimp (*Artemia monica*), the lake’s dominant zooplankter [26]. When grown at low light levels, strain ML exhibits a trilobite morphology [27] reminiscent of Disney’s famous Mickey Mouse [25]. Transcriptomic investigations of the lake’s water column indicated that cryptic photosynthetic activity by strain ML was discernable in the depths beneath, where light could no longer be instrumentally detected [28].

### 2.2. Cell Cultures and Growth of *Picocystis* Strain ML

*Picocystis* strain ML was originally isolated from Mono Lake enrichments by classical dilution/streaking methods at Bigelow Labs National Center for Marine Algae and Microbiota and, when purified, transferred to the USGS in Menlo Park, CA. The strain was cultured in Bigelow L1 seawater-based medium [29] in 500 mL conical flasks for several years after its isolation. Illumination was attained by placing flasks atop a lab bench under general fluorescent lighting (8 h per day) at room temperature (~20 °C). Strain ML tolerates a wide range of salinities, being able to grow from essentially freshwater up to 260 g/L [26]. For the experiments, growth was on one of two phosphate regimens: either kept at the L1 recipe concentration (37 µM) or augmented with 1000 µM KH_2_PO_4_. Experimental variables for both phosphate conditions were the incubation of cells in medium without any arsenic addition or augmented with sodium arsenate (0.8 mM) or sodium arsenite (0.4 mM). Arsenic oxyanion concentrations were determined by HPLC in subsamples taken over the course of the 50–60 days incubations [30], and growth was followed on subsamples by optical density (A_680_). Cells were harvested by centrifugation, washed with sterile L1 medium that lacked arsenic oxyanions, and re-centrifuged. Cell pellets were stored frozen until analyzed by X-ray spectroscopy at the Stanford Linear Accelerator or express-shipped (frozen on dry ice) to the University of Graz, Austria, for the extraction and analysis of arsenic compounds. 

### 2.3. Transmission Electron Microscopy of *Picocystis* Strain ML

Cells were initially fixed in their culture medium with glutaraldehyde (2.5% final concentration), then post fixed (1% osmium tetroxide), dehydrated, and embedded in Spurr’s as described previously [31]. Ultrathin sections were observed on a JEOL 1210 TEM (JEOL, Peabody MA) at 80 kV equipped with an ORCA HR digital camera (Hamamatsu, Bridgewater NJ).

### 2.4. Collection of Field Samples from Mono Lake

Environmental samples were collected in October 2018 at station 6 (lat 37.95, long 119.03 on Mono Lake, CA. A zooplankton net tow (80 µm mesh) was conducted at 0.5 m depth to collect brine shrimp. Discrete water samples were collected at 12, 17, and 20 m depth using a 4-L Niskin closing bottle. Sampling was preceded by a CTD cast (SeaBird SBE 19) to determine water column physical characteristics (e.g., salinity, temperature), light penetration (PAR), and dissolved oxygen concentration. Water was screened (10 µm Nitex) to exclude brine shrimp while filling 2-L Nalgene sample bottles. Water samples were vacuum filtered within several hours of collection using 0.8 µm nylon filters (47 mm diameter). The so obtained plankton samples were frozen and shipped together with the filters. Sediment cores were collected from station 6 using a bottom grab that upon retrieval was sub-cored in duplicate (0–10 cm depth). All samples were frozen on site with dry ice and stored at −80 °C and then shipped frozen to University of Graz for further analysis of arsenic species as outlined below.

### 2.5. Arsenic Species Extractions from Lab-grown *Picocystis* Cells

Extractions of arsenic species were performed in sequence, starting with lipid-soluble arsenicals, followed by aqueous arsenic phases, and finally by a trifluoroacetate (TFA)-based extraction of the “recalcitrant” phase residual after the first two extractions. The non-extractable arsenic remaining in the pellets was solubilized by microwave-assisted acid digestion. Duplicate samples of cells were combined, freeze-dried (0.05 mbar), and the fine powder homogenized with a spatula. For arsenolipids, about 10 mg (weighed to a precision of 0.01 mg) of each sample was transferred into an Eppendorf vial (1.5 mL; polypropylene) in duplicate and extracted with pyridine (>98%; 500 µL) in an ultrasonic bath (15 min at 30 °C) followed by mixing on a rotatory cross (45 min at 20 °C). The suspension was centrifuged (10,000 × g, 10 min., 10 °C) and the liquid phase (ca 450 µL) transferred to a new Eppendorf vial before the remaining pellet was re-extracted with pyridine as described above. The two extracts were combined and lyophilized (10 mbar, 30 °C, 10 h). The dry lipid-extracts were stored (−20 °C) and re-dissolved in pyridine (300 µL) just prior to measurement by reversed phase-high performance liquid chromatography (RP-HPLC) coupled to an inductively coupled plasma mass spectrometer (ICPMS) and simultaneously to an electro spray ionization mass spectrometer (ESMS). The remaining extracted pellets were dried and stored (−20 °C) until the subsequent aqueous extraction was performed.

The post lipid-extraction dried pellets were re-weighed prior to extraction with aqueous buffer. The extractions were performed in the same way as described above except that 20 mM ammonium bicarbonate was employed as the solvent (pH 8.5 adjusted with ammonia; 500 µL). The extracts were dried, stored, and re-dissolved in water (500 µL) just before anion-exchange and cation-exchange HPLC-ICPMS measurements. In order to recover any possible polar water-soluble arsenicals (aqueous As) that were entrained with the arsenolipid extraction, we also performed a liquid/liquid partitioning of the lipid-extract with dichloromethane (DCM)/aqueous buffer, which was also analyzed by HPLC-ICPMS. To do so, a subsample of the re-dissolved pyridine-extract (150 µL out of 300 µL total) was transferred to a new vial, evaporated to dryness (10 mbar, 30 °C, 5 h), re-dissolved in DCM (500 µL), and extracted with aqueous buffer (20 mM ammonium bicarbonate, pH 8.5; 500 µL) on a vortex mixer (1 min, 20 °C). After centrifugation (10,000 × g, 10 min., 10 °C), the water-phase was transferred to a new vial and the procedure repeated with another portion of aq. buffer (500 µL) added to the remaining DCM. The two aqueous phases were combined, evaporated to dryness, stored (−20 °C), and re-dissolved in water (500 µL) just before ion exchange HPLC-ICPMS. Once again, after this analysis the extracted residual pellets were re-dried and stored (−20 °C) until subsequent final extraction with TFA to access the “recalcitrant” arsenic phase (see below).

To access any recalcitrant aqueous As species that resisted the first two extractions, we performed a harsher procedure under acidic aqueous conditions. The pellets were treated with aqueous TFA (1%, v/v; 500 µL) using the same procedure described above (two sequential extractions), and after extraction, the samples were combined, evaporated to dryness, and re-dissolved in water (1 mL) just prior to ion exchange HPLC-ICPMS analysis. The remaining pellets were also lyophilized and stored (−20 °C) until acid digestion.

The final remaining pellets were weighed before microwave assisted digestion with nitric acid (≥65%, 2 × sub-boiled; 2 mL; in closed quartz vessels) and internal standard (aqueous 100 µg/L Ge, In, Te; 1 mL) in an UltraClaveIV microwave digestion system (40 bar argon start pressure; 250 °C for 30 min). The digests were transferred to polypropylene tubes (15 mL) with water and diluted to a final volume of 10 mL before being analyzed by ICPMS. This procedure solubilized the entire pellet, allowing access to the previously non-extractable arsenic remaining in the sample after the three previous extraction steps.

### 2.6. Extraction of Arsenic Species from Collected Field Samples

Upon arrival in Graz, samples were freeze-dried (0.05 mbar). Sediment cores were subdivided into four subsamples in Graz: 0–25, 25–50, 50–75, and 75–100 mm depths. Sediment and *Artemia* samples were homogenized to a fine powder with an agate mortar. About 50 mg of *Artemia*, whole filters containing phytoplankton from each depth (~5 mg dry biomass per filter), and ~200 mg of sediment subsamples (all weighed to a precision of 0.01 mg) were transferred to Eppendorf vials (1.5 mL) in duplicate.

Extractions of arsenic species from collected Mono Lake samples were performed as described above for strain ML but with the following modifications: (i) Lipids were extracted with a mixture of CHCl_3_/EtOH (2 + 1, v/v; 500 µL) containing 1% NH_3_ (v/w); organic extracts of two sequential extractions were combined, dried, and the resultant residue stored (−20 °C) before being re-dissolved in MeOH (500 µL) directly before measurement by RP-HPLC-ICPMS/ESMS. Extracted pellets and sediments were also dried and stored until aqueous extraction. (ii) The re-weighed post lipid-extracted dry pellets and sediments were extracted employing an aqueous ammonia solution (1% NH_3_, v/w; 500 µL), and the combined extracts of two sequential extractions were dried, stored, and re-dissolved in water (1 mL) just before ion exchange HPLC-ICPMS measurements. Extracted pellets and sediments were dried and stored until TFA-extraction. (iii) Liquid/liquid partitioning of the lipid-extracts was performed on subsamples of the re-dissolved alkaline CHCl_3_/EtOH-extract (250 µL out of 500 µL total), which were dried, re-dissolved in CHCl_3_ (500 µL), and extracted with water (500 µL) on a vortex mixer followed by centrifugation. The aqueous layers of two sequential partitions were combined, dried, stored, and re-dissolved in water (1 mL) before ion exchange HPLC-ICPMS. (iv) The remaining pellets and sediments were acid-digested as described for cultured *Picocystis* cells.

We decided to apply two different extraction procedures for arsenolipids: extraction using pyridine for *Picocystis* strain ML or a mixture of CHCl_3_/EtOH under alkaline conditions, which was necessary to release arsenolipids from silica-containing matrices [11,32], e.g., natural Mono Lake plankton collected on nylon filters that contained a SiO_2_-support core. We also used the same two procedures with CRM 7405-a (Hijiki) and obtained no significant differences (p > 0.05 in one-way ANOVA), although we observed a tendency towards slightly lower (ca 5–10%) arsenolipid recoveries when we employed the pyridine extraction method. Nevertheless, extraction with pure pyridine provides several advantages compared to alkaline CHCl_3_/EtOH: first, it obviates the need for alcohols during extraction and reduces the risk of chemical reaction; second, it avoids chlorinated solvents which often cause interferences during ICPMS determinations because of ^35^Cl^40^Ar polyatomic interference on ^75^As; and third, the pyridine extraction method extracts less water-soluble arsenicals. For these reasons, we decided to use the pyridine extraction method when possible.

### 2.7. Chemical Analysis of Extracted Arsenicals

#### 2.7.1. Determination of Arsenolipids

Separation of As species was performed on an ACE Super-Hexyl-Phenyl column (4.6 × 250 mm; 5 µm particles) under gradient elution conditions. This method was adapted from Finke et al. [33]; mobile phase A was water containing 25 mM acetic acid adjusted to pH 9.2 with NH_3_, and B was MeOH containing 25 mM acetic acid and 0.5% NH_3_ (v/v). We used the following gradient: 0–25 min, 40–100% B; 25–35 min, 100% B; 35–35.5 min, 100–40% B; and 35.5–42 min, 40% B. The flow rate was 1.0 mL/min, column temperature was 40 °C, and injection volume was 50 µL. The outflow of the HPLC-system was split (passive splitter) whereby 90% was directed to the ESMS (Agilent 6460) and the remaining 10% transported to the ICPMS (Agilent 7500ce or 7900). Between the splitter and the ICPMS a T-piece was inserted whereby we introduced our internal standard sheath solution (water incl. 0.1 vol% formic acid and 20 µg/L In, Ge, Te; 0.9 mL/min). Carbon compensation [33] was achieved by pumping water/acetone (95 + 5, v/v) directly into the spray chamber with an external rotary pump (0.5 mL/min). The flow to the ESMS subsequently entered the instrument without further alteration. The ESMS was operated in positive SCAN mode (m/z 100–1100) with an applied ionization voltage of 5 kV; 2 kV nozzle voltage; fragmentor voltages were 135 or 200 V; nitrogen gas temperature was 350 °C with a flow of 12 L/min; and the nebulizer pressure was set to 25 psi.

Offline, the extracts were also analyzed by HPLC-high resolution-ESMS/MS using a Q-Exactive Quadrupole Orbitrap mass analyzer equipped with a HESI-II source. The HPLC conditions were adjusted to optimize ionization efficiency, which is considerably higher in positive electrospray ionization mode, when acidic mobile phases are used compared to the alkaline buffers previously applied for HPLC-ICPMS. The applied method was adapted from Glabonjat et al. [22]. The column was a Shodex Asahipak ODP-50 (4.0 × 125 mm; 5 µm particles), and elution was performed using mobile phase A (water containing 0.1 vol% formic acid) and mobile phase B (MeOH containing 0.1 vol% formic acid) with the following gradient: 0–15 min, 60–100% B; 15–23 min, 100% B; 23–23.1 min, 100–60% B; and 23.1–30 min, 60% B. The flow was set to 0.5 mL/min, column held at 40 °C, and injection volume was 10 µL. The instrument was operated in positive ionization mode with 4.2 kV capillary voltage; data dependent MS/MS mode was chosen in m/z window of 120–1200; fragmentation of precursor ions was achieved with N_2_ gas using collision energies of 20, 30, and 40 NCE; and the resolution was 70,000 (FWHM).

#### 2.7.2. Determination of Water-soluble Arsenicals

Cation-exchange HPLC-separations were performed according to Xiong et al. [34] on an IonoSpher 5C column (3.0 × 200 mm; 5 µm particles) under isocratic elution conditions using aqueous 10 mM pyridine (adjusted with formic acid to pH 2.6) as mobile phase. The flow was set to 1.0 mL/min; column temperature was 30 °C; and injection volumes were 5 or 20 µL.

Anion-exchange HPLC-separations of *Picocystis* strain ML extracts were performed after Glabonjat et al. [19] using a PRP-X100 column (4.6 × 150 mm; 5 µm particles) under isocratic conditions with 5 mM malonic acid (adjusted to pH 5.8 with NH_3_) at a flow of 1.0 mL/min; temperature was 30 °C (injection volumes were 20 µL). Field-collected samples were chromatographed on a Thermo AS14A Dionex IonPac column (3.0 × 150 mm, 5 µm particles) under isocratic elution conditions adapted from Narukawa et al. [35] using aqueous 10 mM formic acid (adjusted to pH 4.0 with NH_3_) as mobile phase; flow was 0.7 mL/min; temperature was 30 °C (injection volumes were 5 µL). The outflow of the HPLC-system ran directly into the ICPMS (Agilent 7500ce or 7900). The ICPMS operated in no-gas mode either without optional gas, or with addition of 10 vol% optional gas (Ar/CO_2_, 99 + 1, v/v) for As-signal enhancement.

#### 2.7.3. Determination of Non-extractable As in Digested Pellets

The digested pellets were introduced into the ICPMS (Agilent 7500ce or 7900) through an ASX-500 auto sampler. The ICPMS was operated in the collision mode (5 mL/min He) either without optional gas or with addition of 10 vol% optional gas (Ar/CO_2_, 99 + 1, v/v).

#### 2.7.4. Quality Control of Extracted As Determinations

To monitor the analytical performance and stability of our methods, we used the previously characterized certified reference material (CRM) NMIJ 7405-a (Hijiki) obtained from the Metrology Institute of Japan (Tsukuba, Ibaraki, Japan) under each of the previously described conditions in triplicate. Individual As species (water- and lipid-soluble) were previously determined and are compared in Table S1. We also analyzed an in-house reference material (*Dunaliella tertiolecta*), produced during a previous study in our laboratory in Graz [15], to further validate the applied methods for those As species absent in CRM-Hijiki.

**Figure 1:**
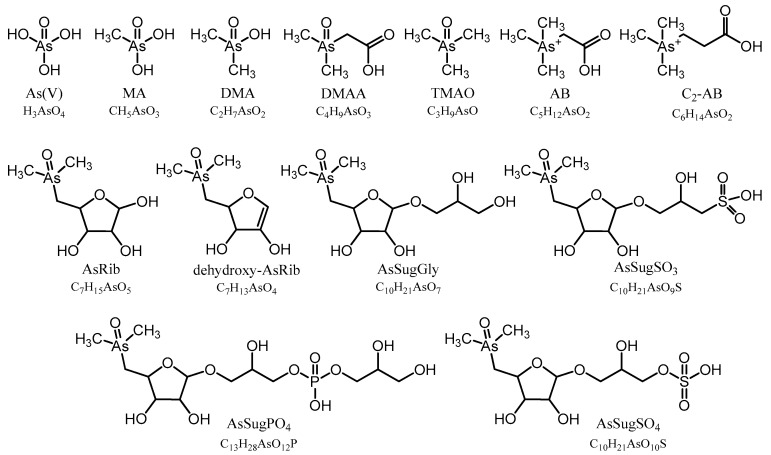
Arsenic containing water-soluble (aqueous As) compounds found in Mono Lake sample extracts.

**Figure 2:**
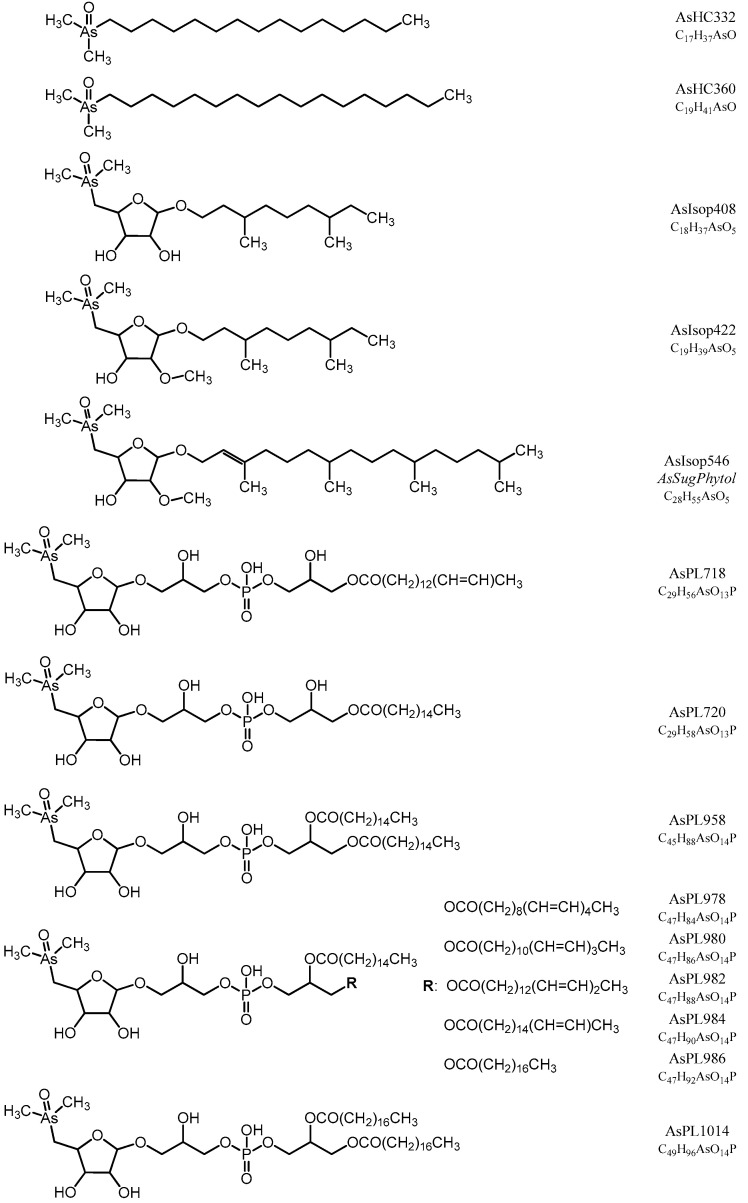
Arsenic containing lipids found in Mono Lake sample extracts. For unsaturated compounds, we show only one possible isomer of the lipophilic side chains. Structures of AsIsop408 and AsIsop422 were proposed based on similarities in their fragmentation spectra with the previously determined phytyl of interest, phytyl 2-*O*-methyl arsenosugar (i.e., AsIsop546 shown above; Figure S1).

To identify individual arsenic species and to monitor the analytical performance and stability of the applied methods, we used retention time matching between standard compounds and environmental samples based on HPLC-ICPMS and HPLC-ESMS for those compounds, where standards were available: As(V), MA, DMA, TMAO, AB, C_2_-AB, AsRib, AsSugGly, AsSugSO_3_, AsSugSO_4_, AsSugPO_4_, AsHC332, and AsHC360. When As standard compounds were not available, we used a combination of the previously determined CRM 7405-a (Hijiki) as a reference for AsPL720, AsPL958, AsPL986, AsPL1014 [32]; and the in-house cultured *Dunaliella tertiolecta* as reference material for AsIsop546, AsPL718, AsPL720, AsPL958, AsPL978, AsPL980, and AsPL982 [15]. We also obtained accurate masses (Δm ≤ 2 ppm) of all identified arsenic species by means of HR-ESMS (Table S2), except for As(V), which did not ionize under the given conditions. In the case of the two newly discovered isoprenoyl 2-*O*-methyl arsenosugars, AsIsop408 and AsIsop422, we also performed fragmentation experiments based on high resolution ESMS and compared the obtained MS^2^ spectra with fragmentation patterns of already known organic arsenicals (Figure S1) to propose the structures of the compounds. All determined arsenic species in this study are shown in Figure 1 and Figure 2.

We used CRM 7405-a (Hijiki) and the in-house cultured reference material *Dunaliella tertiolecta* as samples to assure quantitative results of arsenic species determinations. Total digestion and As determination of CRM 7405-a (Hijiki) provided good agreement with the certified value (35.8 ± 0.9 µg As/g); we measured 35.6 ± 1.0 µg As/g (*n* = 6). The same CRM is also certified for inorganic arsenate (10.1 ± 0.5 µg As/g); we measured 8.5 ± 0.9 µg As/g (*n* = 6), as sum of all aqueous fractions. The lower recovery was possibly related to the slightly different extraction procedure compared with that used in the original CRM determination in which As(V) was directly extracted from dry Hijiki with water at elevated temperatures [36]. Concentrations of other arsenic species than As(V) are not certified in the CRM but can be compared with previously published values (Table S1).

### 2.8. Analysis of Arsenic within *Picocystis* Cells by X-ray Spectroscopy

Bulk X-ray spectroscopy was performed at the Stanford Synchrotron Radiation Lightsource (SSRL) using beam line 7-3. The incident X-ray energy was obtained using a Si (220) double crystal monochromator, with the Stanford Positron Electron Accelerating Ring (SPEAR) storage ring containing 500 mA at 3.0 GeV in top-off mode. Samples were prepared by depositing a small aliquot of the centrifuged pellet onto a nucleopore filter, and the excess moisture of the sample was wicked away with a kimwipe below the filter. Samples were mounted in the beam with a motorized sample positioning system, and the sample position in the incident X-ray beam between each measurement was adjusted slightly to ensure that a fresh part of the sample was analyzed to minimize any beam-induced damage. The incident and transmitted X-ray intensities were measured with nitrogen-filled ion chambers. Energy calibration of the monochromator was monitored using an Au foil measured in transmission geometry between two ion chambers after the sample. Fluorescence detection of As K-alpha fluorescence was measured using a PIPS photodiode detector, when needed for samples containing lower As concentrations. Samples with high As concentrations were processed using the transmission data if possible to reduce the effect of sample self-absorption. Samples were mounted at 45 ° to the incident X-ray beam to minimize scattering. The spectra were collected from 200 eV below the arsenic K-edge to ~1200 eV above the edge, with a minimum counting time of 1 s per point below the edge, up to 30 s per point at the end of the scan. Each scan had a length of approximately 25 minutes. For each sample, approximately 5-20 replicates were measured, depending on the As concentration in the sample, to achieve the desired counting statistics and check for potential beam damage. Spectroscopy data were analyzed using standard methods with the SIXPACK software package [37]. Calculations of the theoretical EXAFS were performed using the FEFF7 package [38].

## 3. Results

### 3.1. Growth of *Picocystis* Strain ML

**Figure 3:**
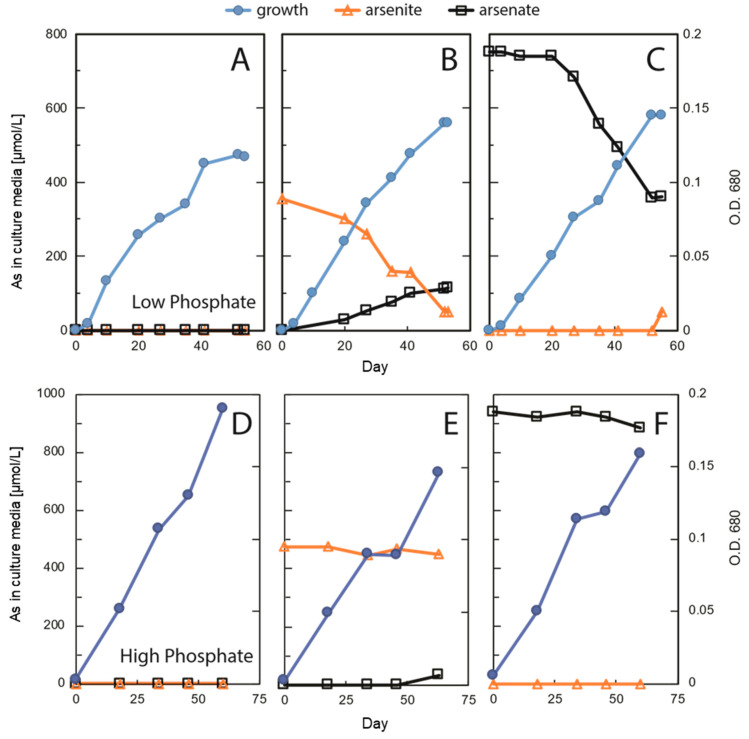
Growth of *Picocystis* strain ML concurrent with As speciation and concentrations in high (1.0 mM) and low (0.037 mM) phosphate media. (**A**) no added As/low P; (**B**) As(III)/low P; (**C**) As(V)/low P; (**D**) no added As/high P; (**E**) As(III)/high P; (**F**) As(V)/high P.

Incubations were conducted over 53 (low P) or 60 days (high P) (Figure 3). Growth in the P-limited samples reached stationary phase by 40 days in controls (Figure 3A), while it continued in As(V)- and As(III)-augmented samples and eventually exceeded that of controls (Figure 3B,C). There was notable loss (~0.3 mM) of As(III) from solution over time that was only partly made up for by recovery as ~0.1 mM As(V), implying that the “missing” arsenic had partitioned into another phase, presumably the biota (Figure 3B). Similarly, there was a comparable loss (~0.3 mM) of dissolved As(V) over time, again with only a partial recovery of ~0.05 mM As(III) evident in solution by the end of the incubation (Figure 3C). We reported similar observations previously for As(V)-incubated strain ML, which, in addition, neither grew nor metabolized As(V) when dark-incubated [25]. Results were different in the P-replete samples, with growth continuing in the As-free controls and eventually reaching higher optical densities (ODs; Figure 3D) than in the P-limited samples (Figure 3A). By comparison, arsenic augmentation tended to slightly constrain growth under these conditions compared to the As-free controls, and there was only a slight loss of As(III) from solution that was eventually recovered as ~0.025 mM As(V) (Figure 3E). There was a only slight loss (~0.05 mM) of As(V) from solution by the incubation’s end, with no notable appearance of As(III) (Figure 3F). However, growth was roughly comparable in all As-augmented samples, whether P-limited (Figure 3B,C) or P-replete (Figure 3E,F). To avoid having the experiment become unwieldy because of too many replicated samples, the experimental conditions represented only one flask per variable. However, the experiment was repeated in subsequent incubations, and the results reported above were replicated (Figure S2).

### 3.2. Electron Microscopy

**Figure 4:**
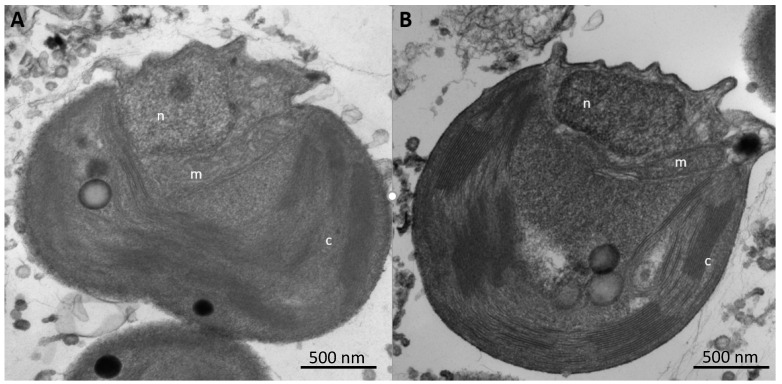
Ultrastructure (TEM) of *Picocystis* strain ML grown under (**A**) phosphorus rich or (**B**) phosphorus poor conditions each in the presence of 0.8 mM As(V). Results suggest ultrastructure was unaffected by As(V) in the medium. n—nucleus, m—mitochondrion, c—chloroplast.

Thin sections of strain ML, whether grown with or without As(V) at low or high P supplementation exhibited comparable fine structures, with their nuclei, chloroplasts, and mitochondria clearly evident (Figure 4A,B). Wider field views of more cells did not reveal any notable structural disparities or impairments between the two populations (Figure S3).

### 3.3. Arsenic Distribution in *Picocystis* Strain ML 

Cultured strain ML cells without added As contained low concentrations of As (~3-4 µg As/g dry wt.), mainly in the form of inorganic As(V) and some non-extractable As species, independent of the P-levels applied (Table 1). This background resulted from our use of the seawater-based L1 medium that contains ~25 nM dissolved arsenic [39]. Extracted As from cultures grown under P-limited conditions with added As contained 1,000-fold higher total As concentrations in As(III) treatments (~3.5 mg As/g dry wt.), and 40,000-fold higher concentrations in As(V) added treatments (~130 mg As/g dry wt.). These bio-accumulated As levels accounted for a large proportion of the dissolved inorganic As that was observed removed from the culture medium over the incubations [e.g., >90% in the dissolved As(V) condition]. A similar pattern was found when strain ML was grown under P-replete conditions, although the effect was less marked with total bio-accumulated As in As(III)-amended cells of ~1 mg As/g dry wt. (300 times control) and ~11 mg As/g dry wt. (3,000 times control) in As(V)-amended cells. Once again, in both cases, most of the dissolved inorganic As removed from the medium was recovered in the microbial biomass [e.g., >70% in dissolved As(V)].

An unexpected finding was the extent of arsenic incorporation into cells incubated under the low P conditions, where it represented as much as 0.35% and 13.3% of their dry weights for the As(III) and As(V) growth conditions, respectively (Table 1). Because a large share of 80–94% (except for low P cultures containing As(III), where it was ca 28%) of this arsenic was associated with the pellet after resisting the first two extractions (Table S3), we suspected it was contained in a recalcitrant macromolecule and conducted further investigations using X-ray spectroscopy to obtain a better characterization of its makeup (see below). Our use of TFA as a stronger extraction reagent resulted in the recovery of most of the arsenic contained in the pellets, releasing it as As(V) (Table S3). In any case, the As species recovered from cells incubated with or without added As(V) were dominated by inorganic As(V), which made up 87–99% of total internal As speciation, independent of the amount of P exposure. In contrast, As(III)-incubated cultures were dominated by non-extractable As (65% of total As) and DMA (60% of total As), under P-limited and P-replete conditions, respectively (Table 1 and Table S3).

**Figure 5:**
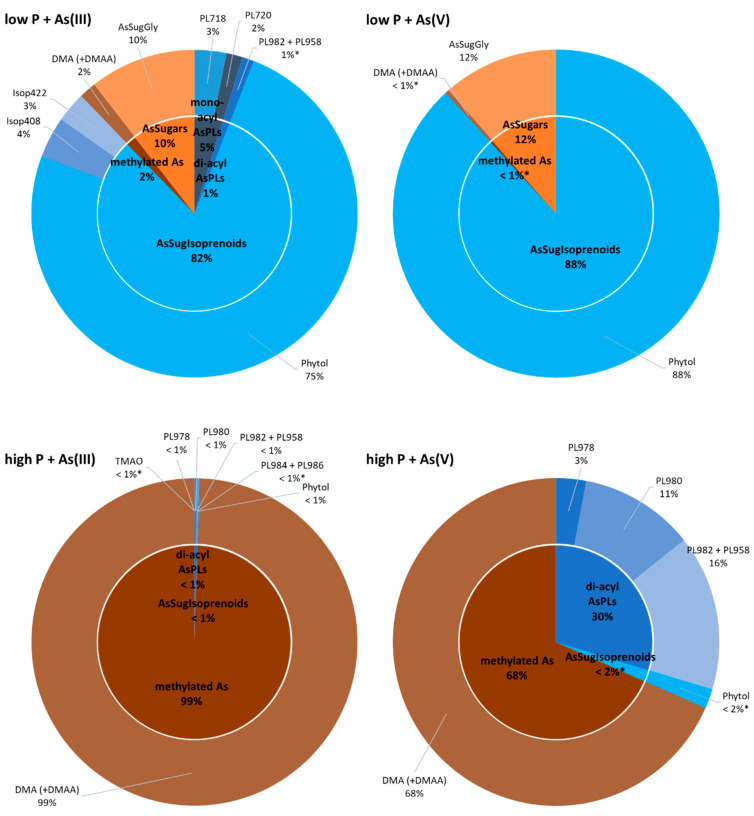
Relative distribution of organic arsenic species in *Picocystis* strain ML cells cultured under various combinations of P-levels and As species (blue: arsenolipids; brown-orange: water-soluble arsenicals). Control cultures of *Picocystis* strain ML contained no detectable concentrations (LOD = 0.1 µg As/g dry mass) of organic As species under both P-levels tested. Detailed quantitative results are presented in Supplementary Table S3 (* indicates < LOQ).

The remaining As species in *Picocystis* strain ML were organoarsenic compounds that we separated into water-soluble (aqueous As) and lipid-soluble (As-lipids) fractions; the molecular structures for these compounds are in Figure 1 and Figure 2, respectively. Their relative distributions in *Picocystis* cells were related more closely to the P-exposure regimen than to the administered As species (Figure 5). Cells cultured under P-limited conditions contained ~90% of the organoarsenic in the form of As-lipids and were dominated by the previously described phytyl 2-*O*-methyl arsenosugar (4–10 µg As/g dry wt., equivalent to 82–88% of the organoarsenic pool; Table S3). Minor arsenic species found in both the As(III) and As(V) amendments were arsenosugar-glycerol (0.5–1.4 µg As/g dry wt.) and dimethylarsinate (DMA) (0.01–0.2 µg As/g dry wt.), both of which are water-soluble. The arsenolipids detected included AsIsop408, AsIsop422, mono-acyl AsPL718, and AsPL720, which were only present in As(III) cultures (Figure 5 and Figure S6 and Table S3) and at comparable concentrations (each 0.2–0.6 µg As/g dry wt.). This picture changed significantly when sufficient P was available to strain ML cells. Under P-replete conditions, the proportion of As-lipids in the cells dropped to less than 1/3 of total organoarsenic, whereas aqueous As (mainly DMA) became the dominant species accounting for 68% (~5 µg As/g dry wt.) or even 99% (~630 µg As/g dry wt.) of all organoarsenic under added As(V) and As(III) conditions, respectively. Besides DMA, we detected minor concentrations of the di-acyl arsenosugar phospholipids AsPL982+958 (1.1–1.5 µg As/g dry wt.), AsPL980 (~0.9 µg As/g dry wt.), AsPL978 (0.2–0.4 µg As/g dry wt.), and traces of AsPL984 + 986 (0.1–0.2 µg As/g dry wt.) and phytyl 2-*O*-methyl arsenosugar (0.1–0.4 µg As/g dry wt.). Quantitative results for individual As species detected under all grown *Picocystis* strain ML culture conditions are summarized in Figure 5 and Table S3.

### 3.4. Characterization of the Recalcitrant Arsenic Pool in *Picocystis* by X-ray Spectroscopy 

**Figure 6:**
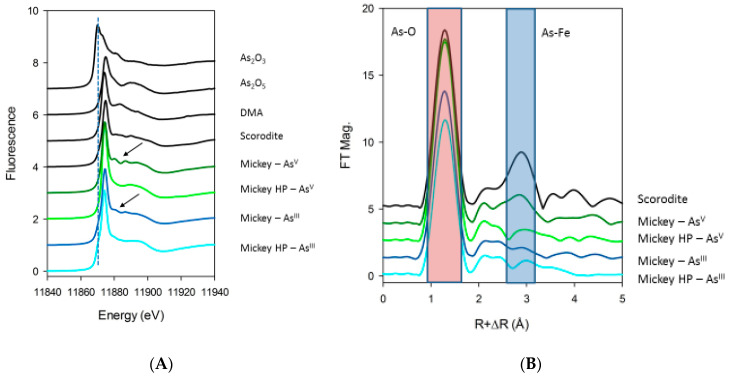
As X-ray spectra of harvested *Picocystis* strain ML (Mickey) samples under various growth conditions compared to standards; (**A**) K-edge XANES and (**B**) K-edge EXAFS. HP—high phosphate cultures.

Cell pellets of the freshly harvested strain ML were examined for the gross chemical speciation of As under each growth condition. Under all growth conditions, the vast majority of As was detected as As(V), i.e., in the pentavalent oxidation state (Figure 6A). The cultures grown in As(III) amended media showed a small shoulder at lower energies, consistent with the presence of As(III). We observed the shoulder in the very first repeat of each condition, being much more prominent in the P-replete system. Successive scans showed that As(III) was oxidized by the incident X-ray exposure in the latter case but had little change in the P-deficient system (Figure S4). The As(III) in these cases is likely due to an amount of As(III) that had been adsorbed onto the surface of the biomass materials and was unable to be released with the centrifuge-wash step on harvesting. Cell pellets harvested from the other growth conditions showed no observable change in arsenic oxidation state during the spectroscopy experiments.

In agreement with the HPLC-ICPMS results, strain ML grown under P-replete conditions showed the presence of methylated arsenic compounds in the pentavalent form. This was indicated by X-ray transition being shifted to lower energies from the inorganic As(V) standard and was very similar to the DMA standard. XAS spectroscopy does not have the ability to distinguish between the complex mixtures of methylated arsenicals, including DMA, arsenosugars, and arsenolipids, yet the resulting XANES spectroscopy is consistent with the pentavalent organoarsenic species. Of note in particular is the As(V) amended, low-P growth condition, which from the As extractions (Table 1) showed the largest concentration of recalcitrant As. This sample also showed prominent resonance features in the post-edge spectra (denoted by arrows in Figure 6A) and a much stronger second-coordination shell backscattering feature in the Fourier-transformed EXAFS (Figure 6B). This feature matches in position with typical As-Fe distances in mixed As-Fe oxide minerals, such as scorodite (FeAsO_4_·2H_2_O), bukovskyite [Fe_2_(AsO_4_)(SO_4_)(OH)·7H_2_O], and pharmacosiderite [KFe_4_(AsO_4_)_3_(OH)_4_·(6-7)H_2_O]. In this *Picocystis* culture, the As EXAFS spectrum does not show as intense a backscattering peak, suggesting that the recalcitrant precipitate is mixed with organoarsenicals, and/or have a higher degree of disorder in the arrangement of the Fe-As mineral structure. The XANES region of the strain ML precipitate is also not a direct match for these model compounds, further suggesting that the resulting recalcitrant material is not directly one of the mineral forms but more likely has a mixed stoichiometry of iron and arsenate.

### 3.5. Arsenic Distribution in Collected Mono Lake Samples

Total As concentrations varied between the different sample types collected, namely mixed plankton (300–500 µg As/g dry wt.), *Artemia* (~120 µg As/g dry wt.), and sediment (60–80 µg As/g dry wt.) (Table 1). In plankton samples, we observed the highest total As at around the density layer (pycnocline) just below the thermocline at 17 m whereas the deeper and shallower samples contained ca 22% and 37% less total As, respectively (Table 1). Sediments collected at four core-sectioned depths showed a slight positive correlation of total As with depth, and we found an As increase of ca 20% from top to bottom sediment layer (Table 1).

**Figure 7:**
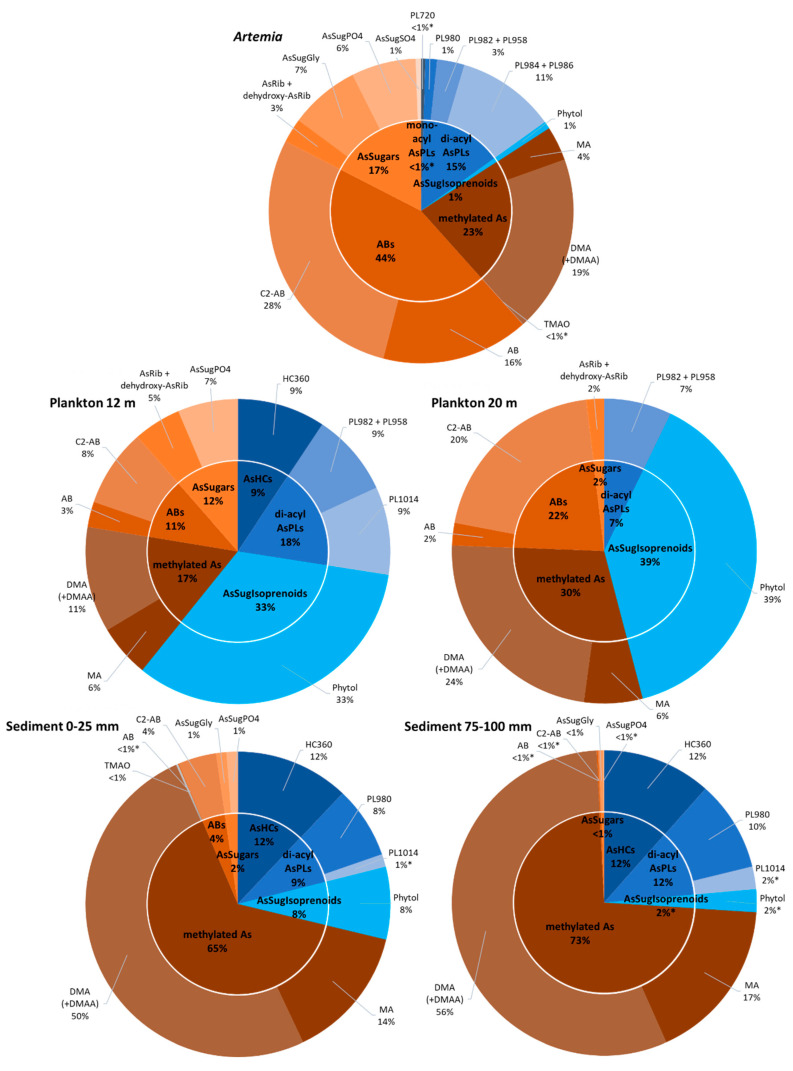
Relative distribution of organic arsenic species in collected Mono Lake samples (blue: arsenolipids; brown-orange: water-soluble arsenicals). Charts for the remaining plankton (17 m) and sediments (25–50 and 50–75 mm) are provided in Figure S5, and detailed quantitative results are presented in Table S4 (* indicates < LOQ).

The major As species in the plankton samples was inorganic As(V), which accounted for ~97% of total As present. These high As(V) concentrations resulted from residual lake water associated with the samples—Mono Lake water contains up to 15 mg As/L (~200 µM) of inorganic As [40]—and thus can mostly be discounted. The remaining extractable As was in the form of As-lipids (45–60%; Figures S5 and S6 and Table S4) and water-soluble organoarsenic species (40–55%), mainly consisting of AsIsop546 (1.1–2.5 µg As/g dry wt.) and simple methylated forms (e.g., DMA and MA; together = 0.6–2.3 µg As/g dry wt.). Minor compounds in plankton were AsPL958+982, C_2_-AB, AB, and AsRib+dehydroxy-AsRib (Figure 7 and Figure S5 and Table S4). Only in the shallowest plankton sample (12 m) did we quantify low concentrations of AsSugPO_4_ (ca 0.25 µg As/g dry wt.) and traces of AsHC360. We recorded the highest concentrations for organoarsenic species in the 17 m depth, except for arsenosugars and arsenobetaines, which were slightly elevated in samples taken above and below (i.e., 12 and 20 m), respectively.

*Artemia* samples showed the largest variety of different As species of all tested samples in this study. As noted above, inorganic As(V) was the major species and accounted for ~90% of total As. From the remaining extractable organoarsenicals, water-soluble As represented ~85% and arsenolipids ~15% (Figure 7 and Table 1). The major water-soluble arsenicals were the arsenobetaines C_2_-AB and AB (together accounting for ~4.5 µg As/g dry wt.), DMA and MA (together ~2.3 µg As/g dry wt.), and the arsenosugars AsSugGly, AsSugPO_4_ and AsRib+dehydroxy-AsRib (together ~1.7 µg As/g dry wt.). As-lipids were mostly di-acyl arsenosugar phospholipids AsPL984+986, AsPL958+982, and AsPL980 (together ~1.5 µg As/g dry wt.; Figure S6). Minor concentrations of AsIsop546 and AsSugSO_4_ were also quantified in *Artemia* together with traces of mono-acyl AsPL720 and TMAO (Figure 7 and Table S4).

Mono Lake sediment samples contained the largest portion of non-extractable As (60–75% of total As; Table 1) of all samples tested. The extractable species were dominated by As(V), which represented 23–39% of the total As and ~95% of all extracted species throughout all four sampled depths. Comparing extractable organoarsenicals, we found higher quantities of water-soluble As (~60–80%) compared to arsenolipids (~20–40%) at all depths. Besides As(V), surface sediments contained As mainly in the form of DMA and MA (together 0.3–0.6 µg As/g dry wt.) and arsenolipid AsHC360 (~0.1 µg As/g dry wt.; Figure S6). We also found lower concentrations of AsIsop546, AsPL980, C_2_-AB, AsSugGly, and AsSugPO_4_ and traces of AsPL1014 and AB (Figure 7 and Figure S5 and Table S4). Although total As increases with depth, we observe a decrease in concentrations of all arsenolipids and most water-soluble organoarsenic species by 30–95% along the core-sectioned sediment depth gradient from surface to 100 mm (Table S4).

## 4. Discussion

Laboratory incubation of *Picocystis* strain ML always generated growth (Figure 3), although not surprisingly, it grew more extensively under high-P conditions than at low-P (Figure 3A,D). Growth at high-P was slightly retarded by inclusion of As(III) or As(V), but there was very little, if any, uptake or redox transformation of the oxyanions under these conditions (Figure 3E,F and Table 1). In contrast, under low-P removal of As(III) was evident as was its partial recovery in solution as As(V) (Figure 3B). Presumably, the “missing” As under this condition was imported into the cells as As(V). In the As(V) culture medium, there was also a notable removal of As(V), but in contrast, there was little recovery in solution as As(III) (Figure 3C). These results were reproducible (Figure S2) and were previously shown to be light dependent [25]. In addition, we noted no changes in sub-cellular structures of cells under these different regimens, indicating that this organism was not experiencing any undue stress due to either P-limitation or As-importation (Figure 4). These observations suggest extensive As uptake into the cells under low-P vs. high-P conditions, as verified quantitatively by the extractions (Table 1). It is also evident that cells were not seriously inconvenienced by this internal As accumulation under the former conditions. Possibly, some of the imported As(V) was employed to substitute for phosphate in various biopolymers, such as membrane associated arsenolipids. This was shown to occur for the bacterium *Agrobacterium tumefaciens* under highly complex cellular regulation [41,42]. It was also observed for the eukaryote thermoacidophile *Cyanidioschyzon* sp., which carried out a variety of arsenic bio-transformations of As-oxyanions, including redox, methylation, and volatilization [43]. Little is known about arsenic metabolism in unicellular eukaryotic algae as compared with prokaryotes, let alone by a *Cyanidioschyzon* sp. isolated from arsenic-rich hot springs. *Picocystis* strain ML, by contrast, is a broadly adaptable haloalkaliphile [26] that is the main photoautotrophic picoplankton inhabiting Mono Lake, an environment particularly rich in arsenic oxyanions (~200 µM). 

Based on our observations (Figure 2) we assume that *Picocystis* strain ML contains the genetic machinery for arsenic uptake (e.g., phosphate transporters; aquaglycerol porins) and for redox transformations (e.g., *arsC; aioBA*) expressed more highly under P-limitation. Such expression occurred in the cyanobacterium *Synechocystis* [44]. A draft genome of strain ML was published [27] but not assembled. Moreover, because the sequences were scattered amongst hundreds of contigs, searches for specifically annotated arsenic genes were impossible to discern at this early stage. What is clear is that strain ML will actively oxidize As(III) to As(V) under P-limitation and will actively acquire As(V) from the aqueous medium under these conditions (Figure 3B,C). This would likely be for its incorporation into macromolecules that under normal P-replete conditions will employ phosphate.

As expected from our above observations, uptake and incorporation of As(V) into cells of strain ML occurred, as evident in the extracted fractions (Table 1). Uptake was most extensive under conditions of P-limitation for both the As(III) [which was oxidized to As(V)] and As(V) amendments, and the lipophilic arsenic portions exceeded those for the P-replete conditions. An unexpected finding was the extensive amount of As recovered from the cells under the low-P conditions. In these cases, the recovered arsenic accounted for 0.35% and 13.3% of the cell dry weight, under the As(III)- and As(V)-treated conditions, respectively. Comparably high As levels were previously only reported in As-hyper-accumulators such as terrestrial ferns containing up to ca 2.7% of their dry mass mainly as inorganic arsenite [45,46]. Most of the here detected algal arsenic was first classified as recalcitrant but ultimately was solubilized as As(V) after TFA extraction, leaving behind smaller amounts of non-extractable arsenic. We initially suspected that this unusually large amount of recalcitrant arsenic was incorporated into a biopolymer, but X-ray spectroscopy revealed it to be primarily a precipitate composed of mixed As-Fe oxide minerals like scorodite (Figure 6) with a smaller quantity of cellular organoarsenic compounds entrained during precipitation. We do not know the specific function of this complex mixture, if any. We speculate that it is an adaptation to the presence of an excessive amount of internal As, which overwhelms the cells’ capacity for methylation and eventual export. It is possible that *Picocystis* strain ML stores this fraction of imported As(V) as a generally inaccessible, innocuous precipitate. Perhaps cells can make use of this faction under sustained nutrient limitation, which if true would make it akin to a luxury uptake of nutrients (i.e., storage) like that for phosphorus and nitrogen [47].

There were broad differences in the composition of organoarsenic species extracted from strain ML under the different incubation regimens (Figure 5). At low-P, most of the extractable As was in the form of arsenolipids, which includes isoprenoyl 2-*O*-metyl arsenosugars, forming the bulk of material recovered, 82% and 88%, from the As(III) and As(V) amended samples, respectively. In the case of the As(III) amendment, most of the arsenic was imported after its external oxidation to As(V), as occurs for *A. tumefaciens* under P-limitation [42]. There was only a small proportion of water-soluble organoarsenic species detected. The opposite situation was clearly evident under P-replete conditions, most strikingly for the As(III) amendment, where arsenolipids made up less than 1% of the extracted organoarsenicals, the remainder being water-soluble As compounds. This also held true for the As(V) amendment, but here arsenolipids represented a much larger percentage fraction (~32%) of the extractable arsenic. We conclude that the imported As(V) likely fulfills some of the functions of phosphate under the conditions of P-limitation. However, the presence of significant amounts of arsenolipids in the high-P/As(V) amendment implies that they can also fulfill a basic cellular function, such as incorporation into the cell’s membrane structure as already suggested by Ender and coworkers [48]. Their near-absence in the P-replete/As(III) amendment suggests that this function is not mandatory for cell growth, and that synthesis of an As(III)-oxidizing protein (e.g., AioBA) is a waste of cellular energy, and its expression is repressed. Curiously, strain ML formed the phytyl 2-*O*-methyl arsenosugar of particular interest in our study, under all added inorganic As incubation conditions. Upregulation of this As-containing potential membrane lipid was obvious under low-P conditions, resulting in ca 30-fold increase in cellular concentrations compared to high-P treatments independent of As-oxyanion amendment (Table S3). Sufficient P supply led to an upregulation of arsenosugar phospholipids (likely incorporated into biological membranes) and, at the same time, complete downregulation of arsenosugars (potential energy storage molecules); the opposite was observed under P-starvation. These observations emphasize the distinct adaptive capacity of *Picocystis* strain ML towards dynamic metabolism of organoarsenic species depending on prevalent environmental challenges.

Owing to the harshness of the lake’s water chemistry (high salinity, high pH, high toxic element concentrations), the ecosystem is simple as there is low diversity in the phytoplankton (primarily *Picocystis*) and zooplankton (primarily *Artemia*) communities, and no vertebrates (i.e., fish) are present. Direct trophic transfer was previously demonstrated between *Picocystis* and *Artemia* in feeding experiments [26]. Arsenolipids were present in all the samples recovered from Mono Lake (Table 1 and Table S3 and Figure 7). Overall, they made up the largest percentage (~60%) of the organoarsenic recovered from the 12 m plankton sample, with the rest composed of water-soluble As, including arsenobetaine. Arsenobetaine is an analog of glycine betaine, and both molecules function as compatible solutes for osmotic balance in this hypersaline environment. The percentage of arsenolipids declined to ~45% in the 20 m plankton sample, while the amount of arsenobetaine doubled, probably due to adaptation to the higher salinity prevalent beneath the thermocline/pycnocline caused by a continuity of lake-wide meromixis (e.g., [49]). Arsenolipids comprised only 16% of the organoarsenic extracted from *Artemia*, while there was a significant arsenobetaine content (44%). This likely reflected the osmoregulatory strategy of this zooplankter, the main consumer of *Picocystis*, thereby gratuitously obtaining a “salt-out” adaptation capacity by feeding. Arsenolipids were still present in the sediment samples, although at lower percentage levels than that displayed in the phytoplankton, presumably due to microbial degradation in the water column and within the sediment itself, as suggested by Glabonjat et al. [22,50]. This is better reflected by the greatly diminished levels of arsenobetaine, which presumably lends itself more readily to anaerobic biodegradation than arsenolipids, analogous to that for glycine betaine [51].

Isoprenoyl 2-*O*-methyl arsenosugars were present (<10%) in the sediment samples and as such now document the occurrence of these unusual compounds, in a new environment besides the oceans [15,52] and the Great Salt Lake [22]. Considering that isoprenoyl 2-*O*-methyl arsenosugars are the only such structures known outside the RNA molecule [15], it is interesting to speculate that they could have had a broader role in the origin of life or geo-biological evolution. The occurrence of these phytol lipids in other environments, such as freshwaters and soils, would indicate a universality and perhaps determine if its relationship to RNA bears any evolutionary significance or is merely coincidental. Considering the surprisingly widespread global occurrence of *Picocystis* strains like *P. salinarium* [53], a closer examination of whether this phytyl 2-*O*-methyl arsenosugar has a broader physiological role than membrane anchoring would be worth investigating.

## Figures and Tables

**Table 1:** Concentrations (µg As/g dry mass) of individual arsenic fractions in *Picocystis* strain ML (Mickey) cultures and collected Mono Lake samples, method blanks, and reference materials. Concentrations are reported as mean ± s. d. of *n* = 2 for *Picocystis* strain ML, *Artemia*, plankton, and sediment; *n* = 6 for blank and CRM Hijiki; and *n* = 4 for *Dunaliella tertiolecta*. The relative distribution of total As is presented in parenthesis as % of total As. Total aqueous As represents the sum of concentrations obtained from alkaline aqueous extraction, aqueous phase of liq/liq partitioning, and TFA-extraction. The relative amounts of total aqueous As obtained from liq/liq-partitioning were always <6% (Mickeys, <2.7%; *Artemia,* ~2.1%; plankton, <0.5%; sediments, <0.3%; Hijiki, ~1.3%; and *D. tertiolecta,* ~5.7%).

	Total As*	Total As-lipids	Total Aqueous As	Total As in HNO_3_-digest
low PO_4_ Mickey control	3.2 ± 0.4	<0.1	2.8 ± 0.3 (88)	0.4 ± 0.2 (12)
low PO_4_ Mickey + As(III)	3,490 ± 100	11.4 ± 0.1 (0.3)	1,280 ± 60 (37)	2,210 ± 80 (63)
low PO_4_ Mickey + As(V)	133,300 ± 1,900	4.2 ± 0.4 (0.003)	126,500 ± 1700 (95)	6,780 ± 340 (5)
high PO_4_ Mickey control	3.4 ± 0.8	<0.1	3.2 ± 0.8 (94)	0.2 ± 0.1 (6)
high PO_4_ Mickey + As(III)	1,030 ± 61	3.2 ± 0.1 (0.3)	1,020 ± 60 (99)	8.2 ± 0.8 (0.8)
high PO_4_ Mickey + As(V)	10,600 ± 200	2.5 ± 0.1 (0.02)	10,600 ± 200 (>99)	27.3 ± 1.1 (0.3)
*Artemia*	123 ± 5	1.6 ± 0.1 (1)	120 ± 5 (98)	1.2 ± 0.1 (1)
Plankton 12 m	312 ± 16	2.5 ± 0.2 (0.8)	302 ± 15 (97)	8.0 ± 0.9 (2)
Plankton 17 m	497 ± 22	2.8 ± 0.1 (0.6)	478 ± 20 (96)	16.1 ± 2.8 (3)
Plankton 20 m	389 ± 52	1.9 ± 0.4 (0.5)	378 ± 51 (97)	8.7 ± 0.6 (2)
Sediment 0–25 mm	63.1 ± 4.2	0.3 ± 0.05 (0.5)	25.2 ± 3.9 (40)	37.6 ± 0.8 (59)
Sediment 25–50 mm	64.5 ± 4.6	0.2 ± 0.05 (0.3)	15.5 ± 0.9 (24)	48.8 ± 4.3 (76)
Sediment 50–75 mm	74.7 ± 4.0	0.1 ± 0.05 (0.1)	17.9 ± 0.2 (24)	56.7 ± 3.9 (76)
Sediment 75–100 mm	74.4 ± 9.9	0.1 ± 0.05 (0.1)	19.9 ± 0.7 (27)	54.4 ± 9.5 (73)
Method blank	<0.2	<0.1	<0.01	<0.05
NMIJ 7405-a CRM (Hijiki)	28.6 ± 2.1	3.8 ± 0.4 (13)	15.4 ± 1.1 (54)	9.4 ± 1.7 (33)
*D. tertiolecta* (Graz)	37.8 ± 2.5	20.3 ± 2.2 (54)	9.6 ± 0.6 (25)	8.0 ± 0.8 (21)

* Total As represents the sum of all three presented fractions.

## Supplementary Materials

The following are available online at https://www.mdpi.com/2075-1729/10/6/93/s1, Table S1. Accuracy of arsenolipid determination based on CRM NMIJ 7405-a (Hijiki) compared to published data. Concentrations are given as µg As/g dry mass. Table S2. High resolution mass spectral data for arsenic species identified in *Picocystis* strain ML and Mono Lake sample extracts in this study. Table S3. Concentrations of individual arsenic species in *Picocystis* strain ML cultures, method blanks, and reference materials tested in this study. Quantification based on HPLC-ICPMS measurements against external calibration with standard compounds. Alkaline and acidic aqueous extracts and aqueous phase of liq/liq-partitioning are summed up; for As(V) and DMA we present the relative fractions found in TFA-extracts in separate lines in *italic* format. Concentrations are given as µg As/g dry mass; limits of detection are represented by ‘< xy’ and limits of quantification by ‘x-y’ (mean ± s.d. of *n* = 6 for blanks; *n* = 2 for *Picocystis* strain ML; *n* = 6 for CRM Hijiki; and *n* = 4 for *Dunaliella tertiolecta*). Table S4. Concentrations of individual arsenic species in collected Mono Lake samples tested in this study. Quantification based on HPLC-ICPMS measurements against external calibration with standard compounds. Alkaline and acidic aqueous extracts and aqueous phase of liq/liq-partitioning are summed up; for As(V) and DMA we present the relative fractions found in TFA-extracts in separate lines in *italic* format. Concentrations are given as µg As/g dry mass; limits of detection are represented by ‘< xy’ and limits of quantification by ‘x-y’ (mean ± s.d. of *n* = 2 for each sample). Figure S1. HR-ESMS/MS of the previously identified phytyl 2-*O*-methyl arsenosugar (AsIsop546) and the newly identified structurally similar arsenolipids AsIsop408 and AsIsop422 isolated from the picoplankter *Picocystis* strain ML. Figure S2. Replicated growth experiments of *Picocystis* strain ML concurrent with As speciation and concentrations in low phosphate (0.037 mM) media. A) no added As; B) As(III) added; C) As(V) added; D & E) these were the samples used for X-ray spectroscopy shown in Figure S3. After centrifugation, pellets were washed with a freshwater medium (Oremland et al., 1994) to avoid interferences from sodium salts. Figure S3. Wider field TEM view of *Picocystis* strain ML cells grown on phosphate with As(V) (left image), or on phosphate without As(V) (right image). Figure S4. Characterization of refractory As in *Picocystis* strain ML by arsenic K-edge XANES. Plots show several repeated measurements at the same location, with the first measurement at the top of the graph, and successive repeats plotted below. (A) As(III) amended system, high P system. (B) As(III) amended system, P-deficient system. The repeats show that the As(III) present in the high P condition is sensitive to beam damage, and is oxidized to As(V) gradually over the course of 3 hours with exposure to X-rays. The As(III) in the P-deficient system and the As(V) amended systems show very little to no change in the spectroscopy over the course of the measurements. Figure S5. Relative distribution of organic arsenic species in collected Mono Lake samples (blue: arsenolipids; brown-orange: water soluble arsenicals). Top, plankton at 17 m depth; and bottom, sediments cores at 25–50 mm and 50–75 mm depths. Detailed quantitative results are presented in Table S4 (* indicates < LOQ). Figure S6. RP-HPLC-ICPMS chromatograms of arsenolipid standards, reference materials, *Picocystis* strain ML cultures, and collected Mono Lake samples. ACE Super-Hexyl-Phenyl column (250 x 4.6 mm; 5 µm particles); gradient elution using mobile phase A: 25 mM ammonium acetate in water, B: 25 mM ammonium acetate in MeOH (both at pH 9.2); flow rate 1 mL/min; column temperature 40 °C; injection volume 50 µL.
