# Supplementary material for: Arsenolipids in Cultured Picocystis Strain ML and Their Occurrence in Biota and Sediment from Mono Lake, California
Source: Life (Basel). 2020 Jun 24;10(6):93. doi: 10.3390/life10060093 (PMC7345539; doi:10.3390/life10060093)
Supplement: Supplementary file 1 [file life-10-00093-s001.pdf]

Supporting Information

Arsenolipids in cultured *Picocystis* strain ML, and their occurrence in biota and sediment from Mono Lake, California.

Ronald A. Glabonjat[a], Jodi S. Blum[b], Laurence G. Miller[b*], Samuel M. Webb[c], John F. Stolz[d], Kevin A. Francesconi[a], and Ronald S. Oremland[b*]

[a] Institute of Chemistry, NAWI Graz, University of Graz, Graz, Austria.

[b] Water Mission Area, US Geological Survey, Menlo Park, California, USA.

[c] Stanford Synchrotron Radiation Lightsource, Menlo, California, USA.

[d] Duquesne University, Pittsburgh, PA, USA.

[*]emeritus

This draft manuscript is distributed solely for purposes of scientific peer review. Its content is deliberative and predecisional, so it must not be disclosed or released by reviewers. Because the manuscript has not yet been approved for publication by the U.S. Geological Survey (USGS), it does not represent any official USGS finding or policy.

Table S1. Accuracy of arsenolipid determination based on CRM NMIJ 7405-a (Hijiki) compared to published data. Concentrations are given as µg As/g dry mass.

**As species**	**This study (*n* = 6)**	**Pétursdóttir et al. 2019 (*n* = 2 As-lipids; *n* = 3 aq. As)**	**Glabonjat et al. 2019 (*n* = 1)**	**Wolle et al. 2018 (*n* = 5)**	**Glabonjat et al. 2018 (*n* = 7)**	**Al Amin et al. 2018 (*n* = 3)**	**Glabonjat et al. 2014 (*n* = 3)**
As(V)	8.51 ± 0.91	*	-	9.1 ± 0.1	10.6 ± 1.2	-	-
MA	0.16 ± 0.01	-	-	-	0.01 ± 0.01	-	-
DMA	1.19 ± 0.39	0.62 ± 0.06	-	0.47 ± 0.02	0.61 ± 0.08	-	-
AsRib + dehydroxy-AsRib	0.64 ± 0.05	-	-	-	-	-	-
AsSugGly	0.25 ± 0.03	0.42 ± 0.05**	-	0.33 ± 0.01	0.60 ± 0.14	-	-
$AsSugSO_3$	0.81 ± 0.25	1.1 ± 0.1	-	0.55 ± 0.04	-	-	-
$AsSugSO_4$	2.66 ± 0.24	14.6 ± 1.2*	-	3.2 ± 0.1	-	-	-
$AsSugPO_4$	1.19 ± 0.03	1.5 ± 0.1	-	1.11 ± 0.03	1.11 ± 0.22	-	-
AsHC332	1.12 ± 0.30	1.1-2.1***	1.45	-	1.07 ± 0.04	1.87 ± 0.17	1.07 ± 0.04
AsHC360	0.1-0.2		0.21	-	0.13 ± 0.02	0.33 ± 0.01	0.09 ± 0.05
AsPL720	0.25 ± 0.09	-		-	-	0.51 ± 0.01	-
AsPL958	1.66 ± 0.23	0.1-2.5	2.47	-	2.41 ± 0.12	2.50 ± 0.30	1.59 ± 0.03
AsPL986	0.36 ± 0.08	0.37 ± 0.09	0.37	-	0.34 ± 0.02	0.54 ± 0.07	0.30 ± 0.01
AsPL1014	0.24 ± 0.04	0.20 ± 0.03	0.3	-	0.23 ± 0.01	0.35 ± 0.05	0.21 ± 0.01
AsPL1042	-	-	0.11	-	0.15 ± 0.01	0.22 ± 0.06	0.110.01

* Concentration as sum of $AsSugSO_4$ + As(V); ** concentration as sum of AsSugGly + arsenite; *** concentration as sum of AsHC332 + AsHC360; - not reported.

Table S2. High resolution mass spectral data for arsenic species identified in *Picocystis* strain ML and Mono Lake sample extracts in this study.

**Compound**	**Elemental composition**	**Calculated mass $[M+H]^+$**	**Measured mass $[M+H]^+$**	**Mass difference [ppm]**
MA	CH_5AsO_3	140.9527	140.9525	-1.7
DMA	$C_2H_7AsO_2$	138.9735	138.9733	-1.3
TMAO	C_3H_9AsO	136.9942	136.9940	-1.5
AB	$C_5H_{11}AsO_2$	179.0048	179.0044	-1.9
C_2-AB	$C_6H_{13}AsO_2$	193.0204	193.0200	-1.8
AsRib	$C_7H_{15}AsO_5$	255.0208	255.0203	-1.8
dehydroxy-AsRib	$C_7H_{13}AsO_4$	237.0102	237.0099	-1.3
AsSugGly	$C_{10}H_{21}AsO_7$	329.0576	329.0573	-0.9
$AsSugSO_3$	$C_{10}H_{21}AsO_9S$	393.0195	393.0193	-0.5
$AsSugSO_4$	$C_{10}H_{21}AsO_{10}S$	409.0144	409.0140	-0.7
$AsSugPO_4$	$C_{13}H_{28}AsO_{12}P$	483.0607	483.0604	-0.3
AsHC332	$C_{17}H_{37}AsO$	333.2133	333.2133	+0.1
AsHC360	$C_{19}H_{41}AsO$	361.2446	361.2449	+0.9
AsIsop408	$C_{18}H_{37}AsO_5$	409.1929	409.1931	+0.2
AsIsop422	$C_{19}H_{39}AsO_5$	423.2086	423.2087	+0.1
AsIsop546	$C_{28}H_{55}AsO_5$	547.3338	547.3340	+0.4
AsPL718	$C_{29}H_{56}AsO_{13}P$	719.2747	719.2747	-0.1
AsPL720	$C_{29}H_{58}AsO_{13}P$	721.2903	721.2906	+0.3
AsPL958	$C_{45}H_{88}AsO_{14}P$	959.5200	959.5201	+0.1
AsPL978	$C_{47}H_{84}AsO_{14}P$	979.4887	979.4891	+0.3
AsPL780	$C_{47}H_{86}AsO_{14}P$	981.5043	981.5039	-0.5
AsPL982	$C_{47}H_{88}AsO_{14}P$	983.5200	983.5193	-0.7
AsPL984	$C_{47}H_{90}AsO_{14}P$	985.5356	985.5366	+0.9
AsPL986	$C_{47}H_{92}AsO_{14}P$	987.5513	987.5520	+0.7
AsPL1014	$C_{49}H_{96}AsO_{14}P$	1015.5826	1015.5836	+1.0

Table S3. Concentrations of individual arsenic species in *Picocystis* strain ML cultures, method blanks, and reference materials tested in this study. Quantification based on HPLC-ICPMS measurements against external calibration with standard compounds. Alkaline and acidic aqueous extracts and aqueous phase of liq/liq-partitioning are summed up; for As(V) and DMA we present the relative fractions found in TFA-extracts in separate lines in *italic* format. Concentrations are given as µg As/g dry mass; limits of detection are represented by '< xy' and limits of quantification by 'x-y' (mean ± s.d. of n = 6 for blanks; n = 2 for *Picocystis* strain ML; n = 6 for CRM Hijiki; and n = 4 for *Dunaliella tertiolecta*).

**As species**	**Blank**	**low P *Picocystis* control**	**low P *Picocystis* +As(III)**	**low P *Picocystis* +As(V)**	**high P *Picocystis* control**	**high P *Picocystis* +As(III)**	**high P *Picocystis* +As(V)**	**NMIJ 7405-a CRM (Hijiki)**	***D. tertiolecta* (Graz)**
As(V)	< 0.01	2.8 ± 0.3	1274 ± 64	126512 ± 1685	3.2 ± 0.8	386 ± 39	10600 ± 220	8.51 ± 0.91	6.93 ±0.43
As(V) in TFA-extract	-	*93 %*	*78 %*	*99 %*	*85 %*	*80 %*	*82 %*	*15 %*	*54 %*
MA	< 0.01	< 0.01	< 0.01	< 0.01	< 0.01	< 0.01	< 0.01	0.16 ± 0.01	< 0.1
DMA	< 0.01	< 0.01	0.22 ± 0.03	0.01-0.03	0.01-0.03	629 ± 46	5.3 ± 7.0	1.19 ± 0.39	0.51 ± 0.03
DMA in TFA-extract	-	-	*< 1 %*	*< 1 %*	-	*94 %*	-	*26 %*	*8 %*
TMAO	< 0.01	< 0.01	< 0.01	< 0.01	< 0.01	0.01-0.03	< 0.01	< 0.1	< 0.1
AB	< 0.01	< 0.01	< 0.01	< 0.01	< 0.01	< 0.01	< 0.01	< 0.1	< 0.1
C_2-AB	< 0.01	< 0.01	< 0.01	< 0.01	< 0.01	< 0.01	< 0.01	< 0.1	< 0.1
AsRib + dehydroxy-AsRib	< 0.01	< 0.01	< 0.01	< 0.01	< 0.01	< 0.01	< 0.01	0.64 ± 0.05	0.1-0.2
AsSugGly	< 0.01	< 0.01	1.37 ± 0.10	0.55 ± 0.03	< 0.01	< 0.01	< 0.01	0.25 ± 0.03	1.47 ± 0.42
$AsSugPO_4$	< 0.01	< 0.01	< 0.01	< 0.01	< 0.01	< 0.01	< 0.01	1.19 ± 0.03	0.51 ± 0.06
$AsSugSO_3$	< 0.01	< 0.01	< 0.01	< 0.01	< 0.01	< 0.01	< 0.01	0.81 ± 0.25	< 0.1
$AsSugSO_4$	< 0.01	< 0.01	< 0.01	< 0.01	< 0.01	< 0.01	< 0.01	2.66 ± 0.24	< 0.1
AsHC332	< 0.1	< 0.1	< 0.1	< 0.1	< 0.1	< 0.1	< 0.1	1.12 ± 0.30	< 0.1
AsHC360	< 0.1	< 0.1	< 0.1	< 0.1	< 0.1	< 0.1	< 0.1	0.1-0.2	0.26 ± 0.08
AsIsop408	< 0.1	< 0.1	0.53 ± 0.02	< 0.1	< 0.1	< 0.1	< 0.1	< 0.1	< 0.1
AsIsop422	< 0.1	< 0.1	0.39 ± 0.03	< 0.1	< 0.1	< 0.1	< 0.1	< 0.1	< 0.1
AsIsop546 (AsSugPhytol)	< 0.1	< 0.1	9.71 ± 0.06	4.24 ± 0.43	< 0.1	0.33 ± 0.01	0.1-0.2	< 0.1	11.23 ± 1.87
AsPL718	< 0.1	< 0.1	0.41 ± 0.06	< 0.1	< 0.1	< 0.1	< 0.1	< 0.1	0.1-0.2
AsPL720	< 0.1	< 0.1	0.21 ± 0.01	< 0.1	< 0.1	< 0.1	< 0.1	0.25 ± 0.09	0.48 ± 0.21
AsPL978	< 0.1	< 0.1	< 0.1	< 0.1	< 0.1	0.37 ± 0.01	0.23 ± 0.02	< 0.1	1.68 ± 0.26
AsPL980	< 0.1	< 0.1	< 0.1	< 0.1	< 0.1	0.88 ± 0.02	0.88 ± 0.05	< 0.1	2.68 ± 0.79
AsPL982 + AsPL958	< 0.1	< 0.1	0.1-0.2	< 0.1	< 0.1	1.48 ± 0.07	1.19 ± 0.03	1.66 ± 0.23	3.79 ± 0.62
AsPL984 + AsPL986	< 0.1	< 0.1	< 0.1	< 0.1	< 0.1	0.1-0.2	< 0.1	0.36 ± 0.08	< 0.1
AsPL1014	< 0.1	< 0.1	< 0.1	< 0.1	< 0.1	< 0.1	< 0.1	0.24 ± 0.04	< 0.1
Total As in HNO_3-digest	< 0.05	0.4 ± 0.2	2206 ± 77	6776 ± 337	0.2 ± 0.1	8.2 ± 0.8	27.3 ± 1.1	9.4 ± 1.7	8.0 ± 0.8

Table S4. Concentrations of individual arsenic species in collected Mono Lake samples tested in this study. Quantification based on HPLC-ICPMS measurements against external calibration with standard compounds. Alkaline and acidic aqueous extracts and aqueous phase of liq/liq-partitioning are summed up; for As(V) and DMA we present the relative fractions found in TFA-extracts in separate lines in *italic* format. Concentrations are given as µg As/g dry mass; limits of detection are represented by '< xy' and limits of quantification by 'x-y' (mean ± s.d. of $n = 2$ for each sample).

**As species**	**Artemia**	**Plankton 12 m**	**Plankton 17 m**	**Plankton 20 m**	**Sediment 0-25 mm**	**Sediment 25-50 mm**	**Sediment 50-75 mm**	**Sediment 75-100 mm**
As(V)	111 ± 5	300 ± 15*	475 ± 20*	376 ± 51*	24.5 ± 3.9	15.2 ± 0.9	17.4 ± 0.2	19.5 ± 0.7
As(V) in TFA-extract	*9 %*	*8 %**	*13 %**	*11 %**	*2 %*	*< 1 %*	*< 1 %*	*1 %*
MA	0.38 ± 0.01	0.21 ± 0.03	0.35 ± 0.02	0.26 ± 0.04	0.13 ± 0.02	0.11 ± 0.01	0.09 ± 0.01	0.09 ± 0.01
DMA	1.95 ± 0.30	0.42 ± 0.08	1.92 ± 0.01	0.98 ± 0.12	0.46 ± 0.04	0.21 ± 0.12	0.35 ± 0.04	0.29 ± 0.03
DMA in TFA-extract	*33 %*	*65 %*	*97 %*	*99 %*	*83 %*	*99 %*	*91 %*	*90 %*
TMAO	0.002-0.005	< 0.02	< 0.02	< 0.02	0.002 ± 0.001	< 0.0005	< 0.0005	< 0.0005
AB	1.62 ± 0.05	0.10 ± 0.02	0.02-0.05	0.10 ± 0.03	0.0005-0.001	< 0.0005	< 0.0005	0.0005-0.001
C_2-AB	2.96 ± 0.08	0.31 ± 0.07	0.39 ± 0.28	0.84 ± 0.29	0.037 ± 0.021	0.007 ± 0.002	0.006 ± 0.001	0.0005-0.001
AsRib + dehydroxy-AsRib	0.27 ± 0.05	0.19 ± 0.01	0.11 ± 0.03	0.08 ± 0.01	< 0.0005	< 0.0005	< 0.0005	< 0.0005
AsSugGly	0.77 ± 0.08	< 0.02	< 0.02	< 0.02	0.010 ± 0.001	0.004 ± 0.001	0.004 ± 0.001	0.002 ± 0.001
$AsSugPO_4$	0.71 ± 0.08	0.24 ± 0.08	< 0.02	< 0.02	0.011 ± 0.003	0.004 ± 0.001	0.002 ± 0.001	0.0005-0.001
$AsSugSO_3$	< 0.002	< 0.02	< 0.02	< 0.02	< 0.0005	0.0005-0.001	0.002 ± 0.001	< 0.0005
$AsSugSO_4$	0.06 ± 0.01	< 0.02	< 0.02	< 0.02	< 0.0005	< 0.0005	< 0.0005	< 0.0005
AsHC332	< 0.02	< 0.2	< 0.2	< 0.2	< 0.005	< 0.005	< 0.005	< 0.005
AsHC360	< 0.02	0.2-0.5	< 0.2	< 0.2	0.11 ± 0.02	0.08 ± 0.01	0.06 ± 0.01	0.06 ± 0.01
AsIsop408	< 0.02	< 0.2	< 0.2	< 0.2	< 0.005	< 0.005	< 0.005	< 0.005
AsIsop422	< 0.02	< 0.2	< 0.2	< 0.2	< 0.005	< 0.005	< 0.005	< 0.005
AsIsop546 (AsSugPhytol)	0.08 ± 0.01	1.25 ± 0.13	2.42 ± 0.02	1.62 ± 0.35	0.07 ± 0.02	0.03 ± 0.01	0.005-0.02	0.005-0.02
AsPL718	< 0.02	< 0.2	< 0.2	< 0.2	< 0.005	< 0.005	< 0.005	< 0.005
AsPL720	0.02-0.05	< 0.2	< 0.2	< 0.2	< 0.005	< 0.005	< 0.005	< 0.005
AsPL978	< 0.02	< 0.2	< 0.2	< 0.2	< 0.005	< 0.005	< 0.005	< 0.005
AsPL980	0.13 ± 0.01	< 0.2	< 0.2	< 0.2	0.07 ± 0.02	0.07 ± 0.03	0.04 ± 0.02	0.05 ± 0.01
AsPL982 + AsPL958	0.30 ± 0.02	0.33 ± 0.10	0.38 ± 0.02	0.30 ± 0.07	< 0.005	< 0.005	< 0.005	< 0.005
AsPL984 + AsPL986	1.09 ± 0.01	< 0.2	< 0.2	< 0.2	< 0.005	< 0.005	< 0.005	< 0.005
AsPL1014	< 0.02	0.2-0.5	< 0.2	< 0.2	0.005-0.02	0.005-0.02	0.005-0.02	0.005-0.02
Total As in HNO_3-digest	1.2 ± 0.1	8.0 ± 0.9*	16.1 ± 2.8*	8.7 ± 0.6*	37.6 ± 0.8	48.8 ± 4.3	56.7 ± 3.9	54.4 ± 9.5

* Some of the determined As(V) might result from the remaining lake water on the filter rather than from the mixed plankton itself.

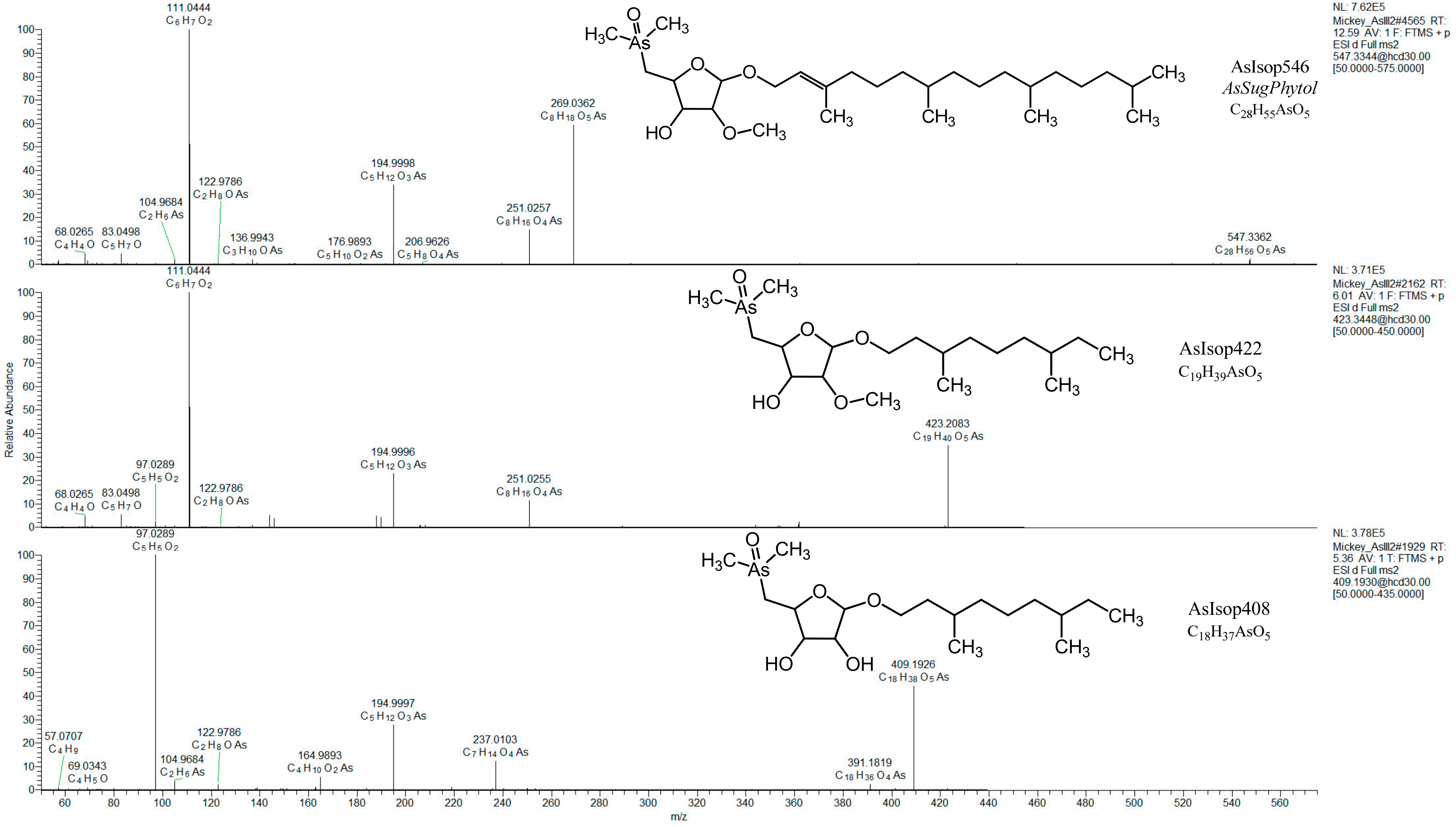

Figure S1. HR-ESMS/MS of the previously identified phytyl 2-*O*-methyl arsenosugar (AsIsop546) and the newly identified structurally similar arsenolipids AsIsop408 and AsIsop422 isolated from the picoplankter *Picocystis* strain ML.

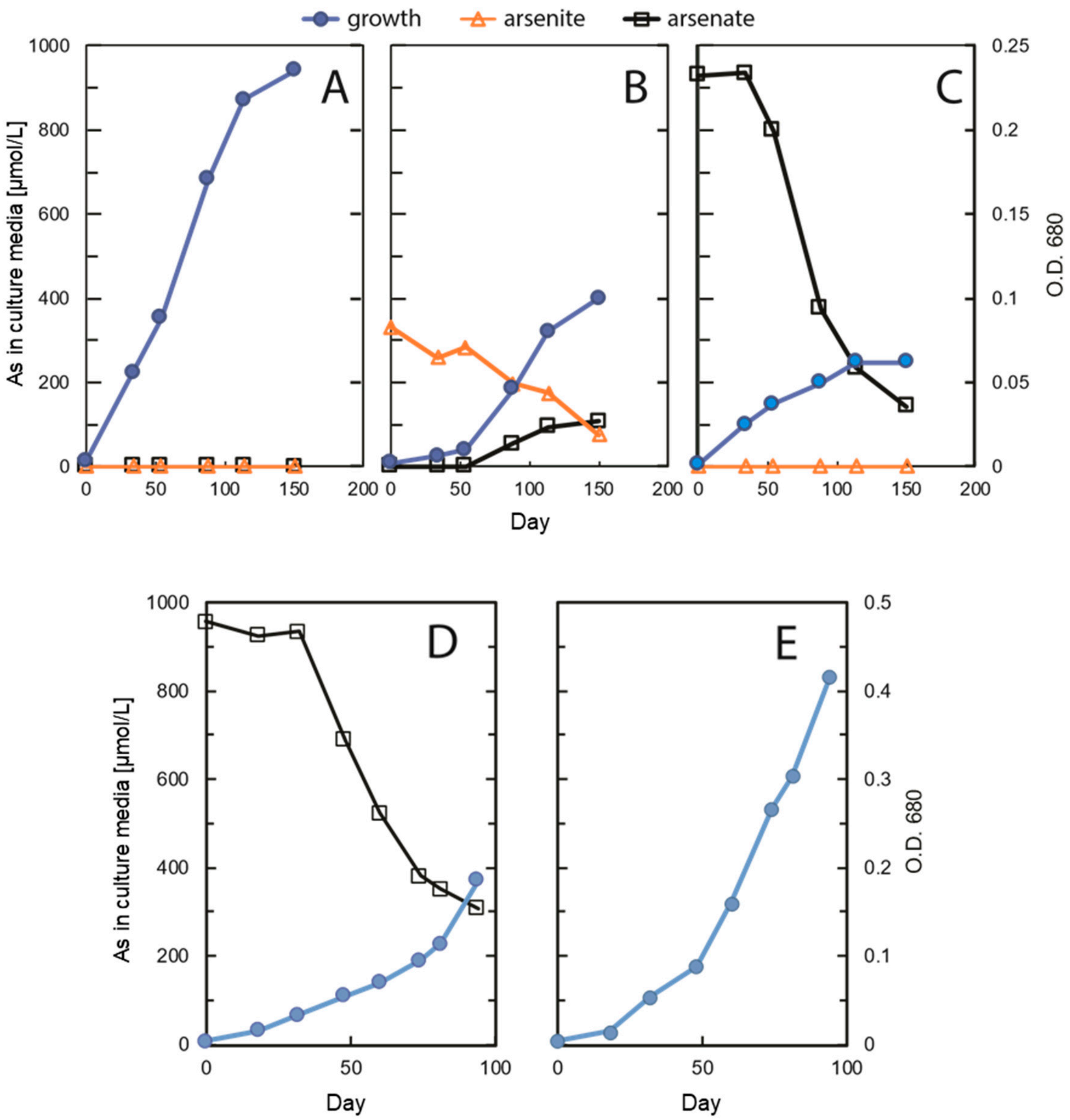

Figure S2. Replicated growth experiments of *Picocystis* strain ML concurrent with As speciation and concentrations in low phosphate (0.037 mM) media. A) no added As; B) As(III) added; C) As(V) added; D & E) these were the samples used for X-ray spectroscopy shown in Figure S3. After centrifugation, pellets were washed with a freshwater medium (Oremland et al., 1994) to avoid interferences from sodium salts.

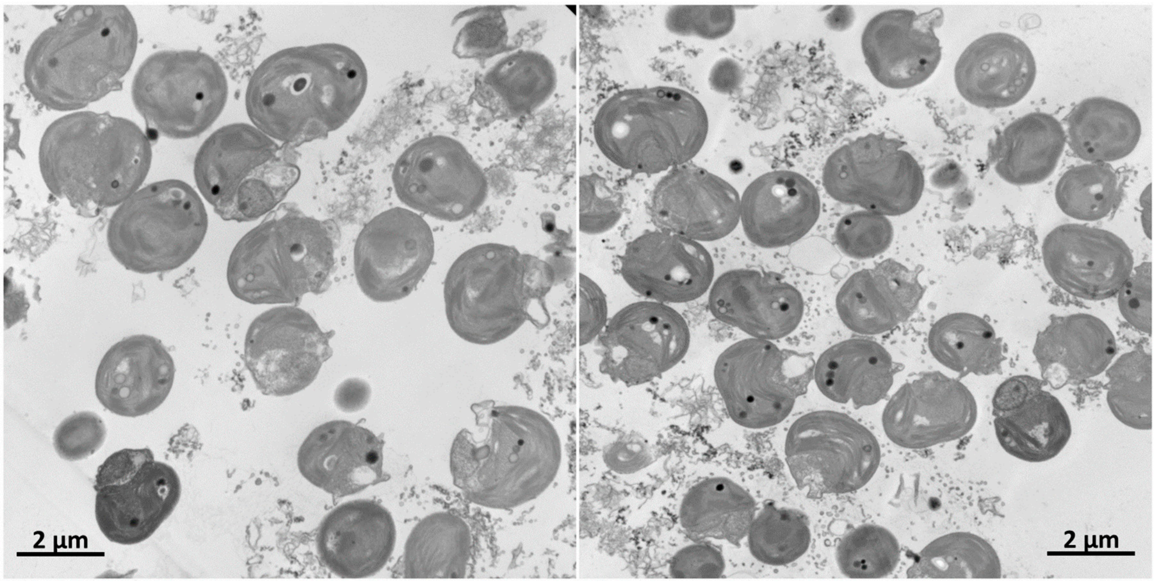

Figure S3. Wider field TEM view of *Picocystis* strain ML cells grown on phosphate with As(V) (left image), or on phosphate without As(V) (right image).

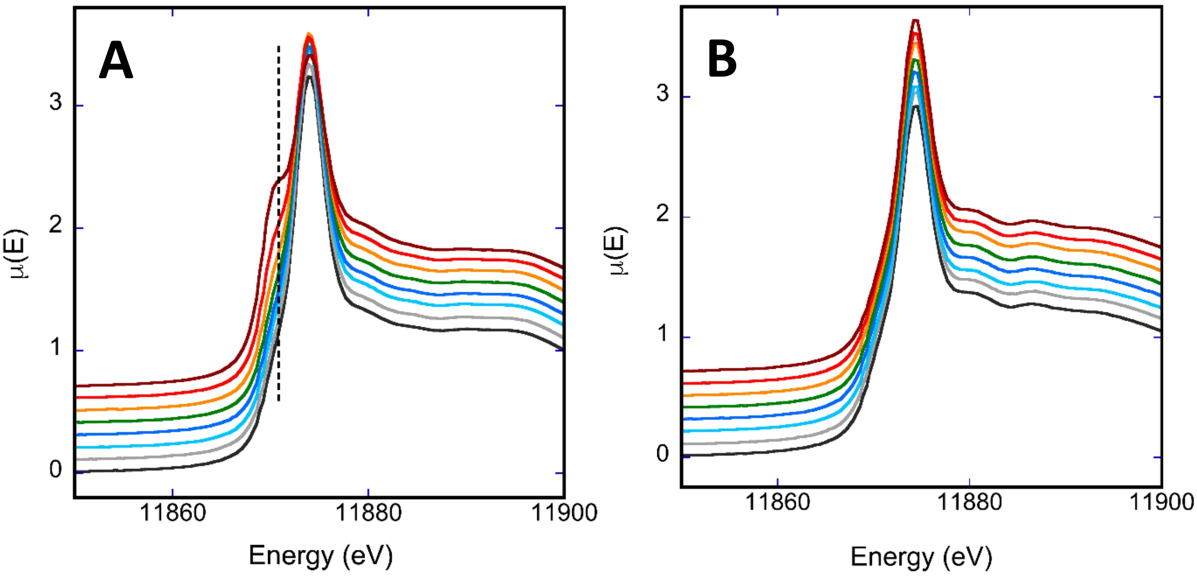

Figure S4. Characterization of refractory As in *Picocystis* strain ML by arsenic K-edge XANES. Plots show several repeated measurements at the same location, with the first measurement at the top of the graph, and successive repeats plotted below. (A) As(III) amended system, high P system. (B) As(III) amended system, P-deficient system. The repeats show that the As(III) present in the high P condition is sensitive to beam damage, and is oxidized to As(V) gradually over the course of 3 hours with exposure to X-rays. The As(III) in the P-deficient system and the As(V) amended systems show very little to no change in the spectroscopy over the course of the measurements.

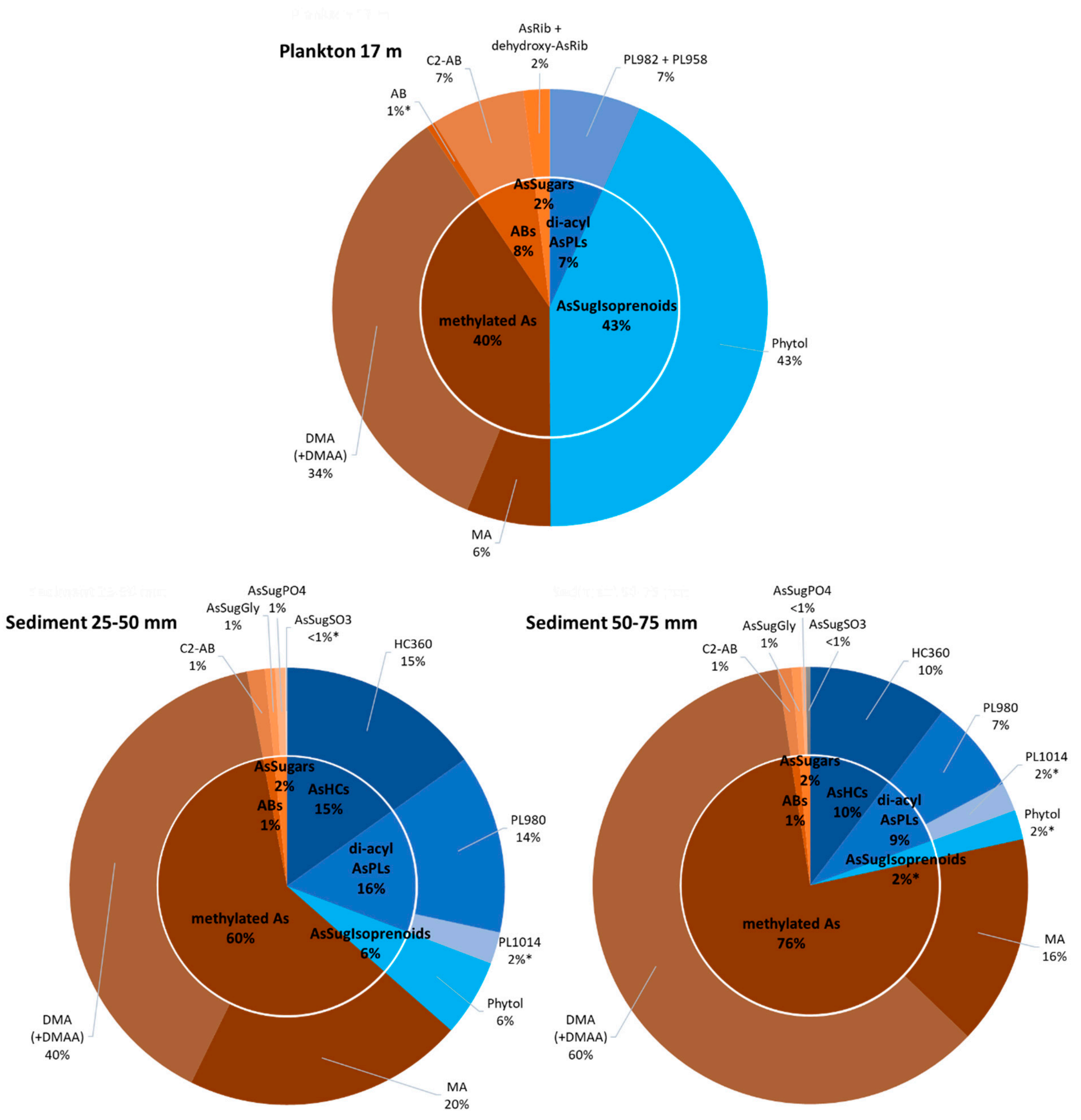

Figure S5. Relative distribution of organic arsenic species in collected Mono Lake samples (blue: arsenolipids; brown-orange: water soluble arsenicals). Top, plankton at 17 m depth; and bottom, sediments cores at 25-50 mm and 50-75 mm depths. Detailed quantitative results are presented in Table S4 (* indicates < LOQ).

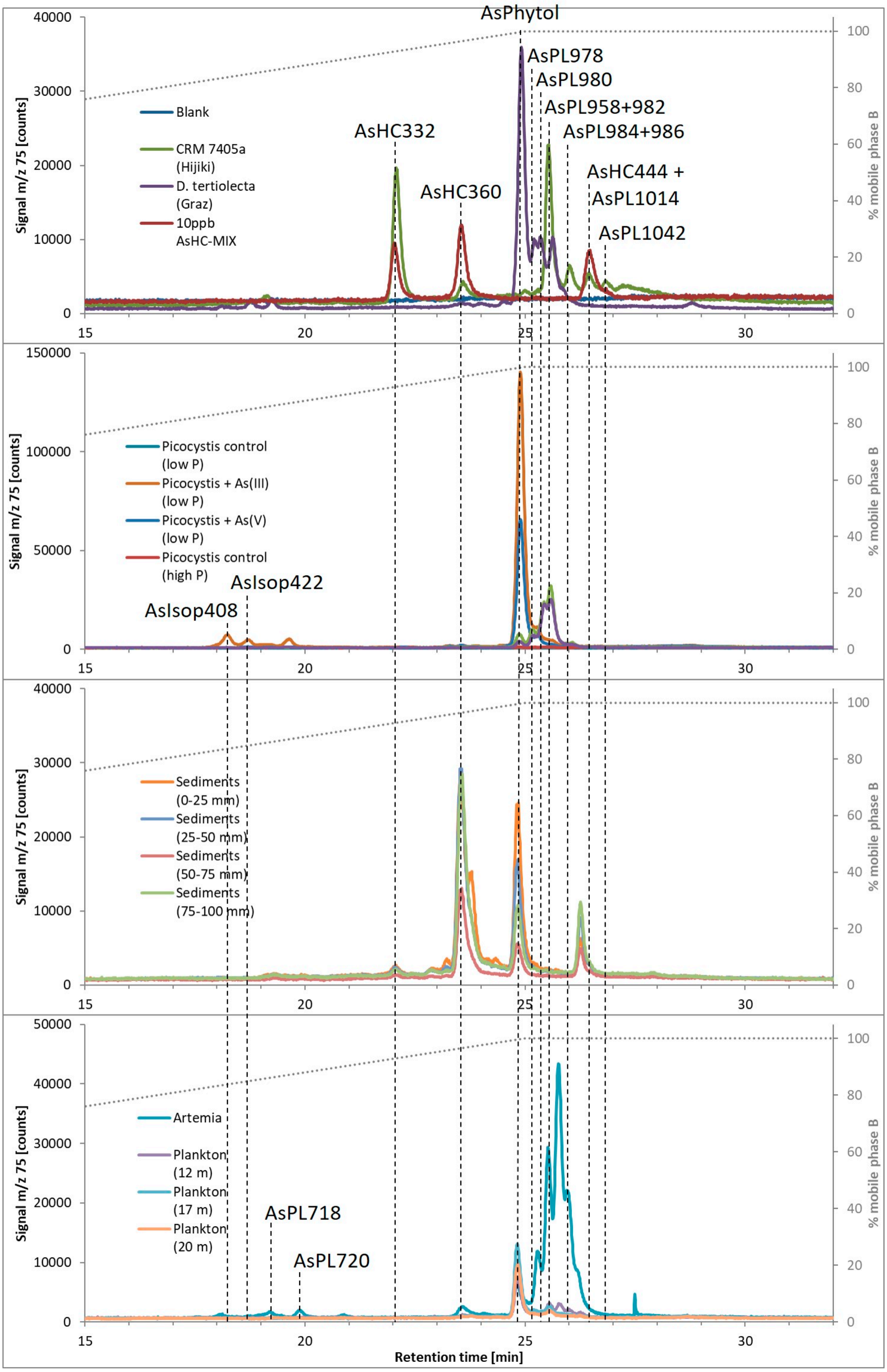

Figure S6. RP-HPLC-ICPMS chromatograms of arsenolipid standards, reference materials, *Picocystis* strain ML cultures, and collected Mono Lake samples. ACE Super-Hexyl-Phenyl column (250 x 4.6 mm; 5 µm particles); gradient elution using mobile phase A: 25 mM ammonium acetate in water, B: 25 mM ammonium acetate in MeOH (both at pH 9.2); flow rate 1 mL/min; column temperature 40 °C; injection volume 50 µL.

A data release report (Blum et al., 2020) can be obtained on-line.

SUPPORTING REFERENCES

Al Amin, M.H.; Xiong, C.; Glabonjat, R.A.; Francesconi, K.A.; Oguri, T.; Yoshinaga, J. Estimation of daily intake of arsenolipids in Japan based on a market basket survey. *Food Chem. Toxicol.* **2018**, *118*, 245–251.

Blum, J.S.; Glabonjat, R.A.; Miller, L.G.; Webb, S.M.; Stolz, J.F.; Francesconi, K.A.; Oremland, R.S.; Baesman, S.M. Growth of cultured Picocystis strain ML in the presence of arsenic, and occurrence of arsenolipids in these Picocystis as well as biota and sediment from Mono Lake, California: U.S. Geological Survey data release, **2020**, https://doi.org/10.5066/P90VW2FP.

Glabonjat, R.A.; Raber, G.; Jensen, K.B.; Ehgartner, J.; Francesconi, K.A. Quantification of arsenolipids in the certified reference material NMIJ 7405-a (Hijiki) using HPLC/mass spectrometry after chemical derivatization. *Anal. Chem.* **2014**, *86*, 10282–10287.

Glabonjat, R.A.; Ehgartner, J.; Duncan, E.G.; Raber, G.; Jensen, K.B.; Krikowa, F.; Maher, W.A.; Francesconi, K.A. Arsenolipid biosynthesis by the unicellular alga Dunaliella tertiolecta is influenced by As/P ratio in culture experiments. *Metallomics* **2018**, *10*, 145–153.

Glabonjat, R.A.; Duncan, E.G.; Francesconi, K.A.; Maher, W.A. Transformation of arsenic lipids in decomposing Ecklonia radiata. *J. Appl. Phycol.* **2019**, *118*, 245.

Oremland, R.S.; Switzer Blum, J.; Culbertson, C.W.; Visscher, P.T.; Miller, L.G.; Dowdle, P.; Strohmaier, F.E. Isolation, growth and metabolism of an obligately anaerobic, selenate-respiring bacterium, strain SES-3. *Appl. Environ. Microbiol.* **1994**, *60*, 3011–3019.

Pétursdóttir, Á.H.; Blagden, J.; Gunnarsson, K.; Raab, A.; Stengel, D.B.; Feldmann, J.; Gunnlaugsdóttir, H. Arsenolipids are not uniformly distributed within two brown macroalgal species Saccharina latissima and Alaria esculenta. *Anal. Bioanal. Chem.* **2019**, *411*, 4973–4985.

Wolle, M.M.; Conklin, S.D. Speciation analysis of arsenic in seafood and seaweed: Part II-single laboratory validation of method. *Anal. Bioanal. Chem.* **2018**, *410*, 5689–5702.

Disclaimer: *Any use of trade, product, or firm names is for descriptive purposes only and does not imply endorsement by the U.S. Government.*
